# Axillary buds are dwarfed shoots that tightly regulate GA pathway and GA-inducible 1,3-β-glucanase genes during branching in hybrid aspen

**DOI:** 10.1093/jxb/erw352

**Published:** 2016-10-03

**Authors:** Päivi L.H. Rinne, Laju K. Paul, Jorma Vahala, Jaakko Kangasjärvi, Christiaan van der Schoot

**Affiliations:** ^1^Department of Plant Sciences, Norwegian University of Life Sciences, N-1432 Ås, Norway; ^2^Division of Plant Biology, Department of Biosciences, University of Helsinki, FI-00014 Helsinki, Finland; ^3^College of Science, King Saud University, Riyadh 11451, Saudi Arabia

**Keywords:** Apical dominance, axillary branching, bud dormancy, callose, gibberellin, 1,3-β-glucanase, para-dormancy, plasmodesmata, Populus, strigolactone.

## Abstract

Axillary buds (AXBs) of hybrid aspen (*Populus tremula*×*P. tremuloides*) contain a developing dwarfed shoot that becomes para-dormant at the bud maturation point. Para-dormant AXBs can grow out after stem decapitation, while dormant AXBs pre-require long-term chilling to release them from dormancy. The latter is mediated by gibberellin (GA)-regulated 1,3-β-glucanases, but it is unknown if GA is also important in the development, activation, and outgrowth of para-dormant AXBs. The present data show that para-dormant AXBs up-regulate GA receptor genes during their maturation, but curtail GA biosynthesis by down-regulating the rate-limiting *GIBBERELLIN 3-OXIDASE2* (*GA3ox2*), which is characteristically expressed in the growing apex. However, decapitation significantly up-regulated *GA3ox2* and GA_4_-responsive 1,3-β-glucanases (GH17-family; α-clade). In contrast, decapitation down-regulated γ-clade 1,3-β-glucanases, which were strongly up-regulated in maturing AXBs concomitant with lipid body accumulation. Overexpression of selected GH17 members in hybrid aspen resulted in characteristic branching patterns. The α-clade member induced an acropetal branching pattern, whereas the γ-clade member activated AXBs in recurrent flushes during transient cessation of apex proliferation. The results support a model in which curtailing the final step in GA biosynthesis dwarfs the embryonic shoot, while high levels of GA precursors and GA receptors keep AXBs poised for growth. GA signaling, induced by decapitation, reinvigorates symplasmic supply routes through GA-inducible 1,3-β-glucanases that hydrolyze callose at sieve plates and plasmodesmata.

## Highlight

Axillary buds uniquely regulate gibberellin (GA) pathway genes, enabling them to stay inhibited but simultaneously poised for growth. Decapitation promotes expression of GA-inducible 1,3-β-glucanase genes that function to reinvigorate symplasmic connections to the stem.

## Introduction

The architecture and three-dimensional shape of a tree arise gradually during multiple seasons. Branches compete for resources, whereas the development of secondary, tertiary, and higher order branches exponentially enhances the complexity of a tree (Turnbull, 2005; Costes *et al.*, 2014; Costes and Gion, 2015). Branching patterns are constrained by phyllotaxis as branches grow out of axillary meristems (AXMs) that arise in the axils of leaves. Although the molecular mechanisms that regulate branching are thought to be conserved between annuals and woody perennials, there are some distinct differences in the initiation of AXMs, axillary bud (AXB) formation, and branching. For example, in the annual Arabidopsis, AXM initiation is delayed, and the AXBs they produce are simple and lack scales (Grbic and Bleecker, 2000; Long and Barton, 2000; Greb *et al.*, 2003). In contrast, deciduous woody perennials produce AXMs by default, in conjunction with nascent leaves, and in continuity with the shoot apical meristem (SAM) (Garrison, 1955). The first products of these AXMs are bud scales which confine an emerging dwarfed shoot (Romberger, 1963; Brunner *et al.*, 2014; Rinne *et al.*, 2015). Differences in the timing of AXB outgrowth give rise to distinct branching styles. In the proleptic branching style, AXBs must pass through a dormancy phase before they can grow out (Hallé *et al.*, 1978; Barthélémy and Caraglio, 2007). In contrast, in sylleptic branching, incomplete AXBs give rise to branches (Wu and Hinckley, 2001). Whereas syllepsis is highly variable due to its environmental responsiveness, the more robust phenomenon of prolepsis reflects strong apical dominance (Ceulemans *et al.*, 1990; Cline, 1997; Wu and Stettler, 1998).

Apical dominance is the phenomenon whereby AXBs are held captive in a state of para-dormancy by a proliferating apex (Phillips, 1975; Cline, 1991, 1997). AXBs can be released from this repressed state by decapitation, a procedure that has been widely used to study branching. Early studies attributed the dominance of the apex (i.e. its developmental supremacy over the AXBs) to the local production of auxin and its basipetal transport (Thimann and Skoog, 1934; Phillips, 1975; Cline, 1991). In a recent model for Arabidopsis, the apex monopolizes the polar auxin transport stream (PATS), saturating its transport capacity and hampering access of AXBs to the PATS (Domagalska and Leyser, 2011). The concept of auxin-based apical dominance is relative in the sense that a proliferating apex does not always prevent branching (Dun *et al.*, 2006). Moreover, in caulescent plants, AXBs may be activated ahead of the decapitation-induced depletion of the PATS (Morris *et al.*, 2005), a phenomenon that was attributed to the sudden availability of sucrose (Mason *et al.*, 2014). This conclusion is reminiscent of the early hypothesis that the growing apex deprives the AXBs of nutrients (Cline, 1991), and suggests that resource allocation is a factor in apical dominance. The emerging picture is that the role of auxin in branching is not straightforward as branching is sensitive to the overall balance of systemic and local processes. In brief, the systemic networks involve auxin, the branch inhibitor strigolactone, the branch facilitator cytokinin, as well as competition-driven shifts in sink–source relationships (Ferguson and Beveridge, 2009; Domagalska and Leyser, 2011; Mason *et al.*, 2014). In addition to the systemic networks, AXBs may self-regulate through internally produced agents. In Arabidopsis, such a local agent is BRANCHED1 (BRC1), a homolog of the maize transcription factor teosinte branched1 (Doebley *et al.*, 1997). BRC1 acts downstream of strigolactone to suppress AXB outgrowth (Aguilar-Martinez *et al.*, 2007; Niwa *et al.*, 2013). Activation of an AXB involves initiation of auxin biosynthesis, production and polarization of PINFORMED1 (PIN1) auxin efflux carriers in the bud-to-stem path, and differentiation of functional vascular connections to the main stem (Li and Bangerth, 1999; Balla *et al.*, 2011; Domagalska and Leyser, 2011).

Deciduous woody perennials may recruit similar mechanisms as their response to decapitation is essentially the same (Cline, 1991, 1997; Rinne *et al.*, 1993), and their genomes contain genes homologous to those involved in herbaceous branching (Czarnecki *et al.*, 2014; Waldie *et al.*, 2014). For example, in hybrid aspen (*Populus tremula*×*P. tremuloides*), *BRC1* and *MAX1* were identified (Rinne *et al.*, 2015). Both genes appeared to be highly expressed in AXBs and down-regulated upon decapitation. Moreover, xylem feeding of the synthetic strigolactone analog GR24 inhibited AXB activation in internode cuttings (Rinne *et al.*, 2015). Together this indicates that they can function locally in AXBs. In trees, such local agents might be particularly important considering the extended transport paths.

In the hybrid aspen clone T89, apical dominance prevents branching in current year AXBs, but it does not prevent the development of an embryonic shoot, which is essentially a dwarfed side shoot. The newly formed AXM follows a developmental program, in which it first produces five primordia that develop into ‘perfect’ scales to protect the subsequent 10 primordia that develop into embryonic leaves. AXB development is completed at the so-called bud maturation point (BMP) (Rinne *et al.*, 2015), and results in a para-dormant ‘embryonic’ or ‘pre-formed’ shoot (Romberger, 1963; Brunner *et al.*, 2014; van der Schoot *et al.*, 2014; Rinne *et al.*, 2015). During branching, the tightly packed embryonic leaves expand, the compressed internodes elongate, and subsequently new leaves are initiated. Thus, in proleptic branching, cell division and morphogenesis of the branch are temporarily separated from expansion by a waiting period of minimally one season.

The dwarfed stature of the embryonic shoot suggests that AXBs are gibberellic acid (GA) deficient. Nonetheless, the role of GA in apical dominance and para-dormancy has not received much attention. One reason might be that the phenotypes of GA biosynthesis mutants (Murfet and Reid, 1993; Silverstone *et al.*, 1997) and overexpressors of GA catabolism genes often show enhanced branching, in both herbaceous plants and trees (Agharkar *et al.*, 2007; Lo *et al.*, 2008; Mauriat *et al.*, 2011; Zawaski and Busov, 2014). This seems to suggest that GA actually inhibits branching (Scott *et al.*, 1967; Rameau *et al.*, 2015). On the other hand, overexpression of GA catabolism genes has variable effects on branching (Busov *et al.*, 2003; Mauriat *et al.*, 2011). This might reflect the complex feed-back structure of the GA pathway (Hedden and Thomas, 2012), with branching depending on the balance of GA biosynthesis, catabolism, and receptor abundance (Ueguchi-Tanaka *et al.*, 2005).

Most overexpression studies focus on GA levels in the stem, while the stem and AXBs might respond independently. The importance of GA levels in AXBs themselves is demonstrated by the up-regulation of GA biosynthesis genes in AXBs of hybrid aspen that are released from dormancy by chilling (Rinne *et al.*, 2011). In support of this, GA supply also induces branching in a number of woody species (Saure, 1985; Rinne *et al.*, 2011; Ni *et al.*, 2015). AXBs of hybrid aspen resemble short day (SD)-induced terminal buds (TBs), both morphologically and molecularly (Rinne *et al.*, 2015), although their vascularization might initially differ (Pizzolato and Larson, 1977). In TBs, dwarfing and eventual cessation of development involve the narrowing of plasmodesmata (PD) and their subsequent closing during dormancy establishment (Rinne and van der Schoot, 1998; Ruonala *et al.*, 2008). This is achieved by local activation of 1,3-β-glucan synthase (glycosyl transferase GT48-family), an enzyme that deposits callose in dormancy sphincter complexes at the PD of the SAM (Ruonala *et al.*, 2008). Such precisely regulated disruption of symplasmic circuitry by ‘circuit breakers’ (Paul *et al.*, 2014*a*) effectively prevents metabolic and electric coupling, and the movement of transcription factors and morphogens that sustain SAM function (Rinne and van der Schoot, 1998; Rinne *et al.*, 2001; Kim *et al.*, 2003; Urbanus *et al.*, 2010). The central role of PD is also evident from experiments in Arabidopsis, in which artificially induced callose at the PD of the SAM compromised or terminated SAM function (Daum *et al.*, 2014). It is likely, therefore, that symplasmic alterations are also instrumental in regulating AXB development and branching.

Symplasmic permeability is under homeostatic regulation by 1,3-β-glucan synthases and callose-hydrolyzing 1,3-β-glucanases (glycosyl hydrolase GH17) at PD and sieve plate pores (Rinne and van der Schoot, 2003; Levy *et al.*, 2007; Levy and Epel, 2009). During dormancy establishment, the balance shifts toward net callose deposition, whereas chilling-induced release reverses it. In hybrid aspen, the central 1,3-β-glucanases involved are GA responsive (Rinne *et al.*, 2011). Xylem feeding of GA and chilling of dormant AXBs affect the local expression of these callose-degrading enzymes, indicating that GA and 1,3-β-glucanases are players in dormancy release and branching.

GH17-families are relatively large in both Arabidopsis (~50 members) and *Populus trichocarpa* (~100). Family members are grouped into three clades (Doxey *et al.*, 2007; Rinne *et al.*, 2011), 10% of which have a cell wall-related function (Geisler-Lee *et al.*, 2006; Doxey *et al.*, 2007). Shoot elongation mostly involves α-clade members, whereas γ-clade members function prominently in defense and stress responses (Maule *et al.*, 2011; Rinne *et al.*, 2011; Benitez-Alfonso *et al.*, 2013; Sager and Lee, 2014). Many α-clade members possess a glycosylphosphatidylinositol (GPI) anchor for attachment to the exoleaflet of the plasma membrane, and/or a carbohydrate-binding module 43 (CBM43) that binds cell wall callose (Doxey *et al.*, 2007; Levy and Epel, 2009; Simpson *et al.*, 2009). The more distant γ-clade members lack known signals and may associate with lipid bodies (Rinne *et al.*, 2011). In Arabidopsis, members of the α-clade localize to PD (Levy *et al.*, 2007; de Storme and Geelen, 2014; Gaudioso-Pedraza and Benitez-Alfonso, 2014; Knox and Benitez-Alfonso, 2014). In hybrid aspen, both α- and γ-clade members can localize to PD, potentially targeting callose deposits at distinct parts of the PD (Paul *et al.*, 2014*b*).

Here, we investigate the putative roles of GA pathway and GH17-family genes in embryonic shoot dwarfing, para-dormancy, dormancy, and branching. The results suggest that AXBs are GA deficient but highly sensitive to GA owing to low expression of the rate-limiting GA biosynthesis gene *GIBBERELLIN 3-OXIDASE2* (*GA3ox2*) and high expression of two *GIBBERELLIN INSENSITIVE DWARF1*-like (*GID1*-like) GA receptor genes. The rate-limiting *GA3ox2* is significantly up-regulated in AXBs after decapitation, but not in decapitation-insensitive dormant AXBs, showing that SDs block its expression whereas chilling de-represses it. Expression analyses of GA-responsive α- and γ-clade members of the GH17-family indicate that these enzymes modulate symplasmic permeability during AXB transitions. Functional studies in which representative members of the α- and γ-clade were overexpressed in hybrid aspen support the conclusion that GA biosynthesis, and its downstream effects on GH17-family members, are crucial in AXB formation and activation.

## Materials and methods

### Plant material and designs for experiments

Hybrid aspen (*Populus tremula*×*P. tremuloides*) clone T89 was micropropagated *in vitro*, planted in soil as described previously (Ruonala *et al.*, 2008), grown in a greenhouse under long days (LDs) (18h light) at ~18 °C and 75–80% relative humidity, and watered twice a day. Natural light was supplemented to a level of 200 µmol m^−2^ s^−1^ at 400–750nm (Osram). After 6 weeks, when the plants had reached a height of 70–80cm, and elongation and leaf production rates were constant, the plants were divided into three groups. Group one was kept in LDs as a control group. Group two was moved to an SD regime with a 10h photoperiod for 5 weeks to induce dormancy. Group three was kept in LDs and decapitated just above the BMP to remove apical dominance. Kinetics of AXB outgrowth and dormancy were described previously (Rinne *et al.*, 2015).

Initiation of SD-induced TBs is strictly controlled by photoperiod length and requires repeated photoperiodic cycles. To compare AXBs and TBs of the same developmental age, it was necessary to determine how many SD cycles were required to initiate TBs. As no molecular markers of TB initiation are known, and the early morphological changes are hidden from sight by a cluster of newly initiated leaves, we used two indirect growth analyses methods. First, we counted the number of nascent leaves and primordia around the SAM of an LD plant, and assessed how many of these leaves unfolded under SDs before the TB became visible, which took 21–28 d. With the second growth analysis, we established where in this trajectory the first signs of irreversible TB formation could be detected in plants that were returned to LDs.

### AXB anatomy and staining of lipid and callose

AXBs were fixed overnight at 4 °C in 2% (v/v) glutaraldehyde and 3% (v/v) paraformaldehyde in 100mM phosphate citrate buffer, as described earlier (Rinne *et al.*, 2001). Briefly, samples were infiltrated gradually with LR White Resin (LRW) of increasing concentration (30–70%), and kept for 4 d in 100% LRW. Polymerization was conducted at 55 °C for 24h. Lipid body accumulation was studied by staining longitudinally cut 1–3 µm thick sections with Sudan II (black) (1% w/v in 70% ethanol), filtered prior to use. The sections were stained in a continuously stirred solution at 50 ^o^C, subsequently cleared with 70% ethanol for 2min, and mounted in water for light microscopic observation. For callose staining, longitudinal fresh hand sections were made under a dissection microscope through AXB–node units that were submersed in 10mM 2-deoxy-d-glucose (2-DDG; Sigma-Aldrich) to inhibit formation of cutting-induced callose (Rinne *et al.*, 2005). Subsequently, the sections were incubated in the dark for ~1h in 0.1M K_2_HPO_4_ buffer (pH 9.5) containing aniline blue (0.01%) and 2-DDG (5mM). Callose deposits were examined with epi-fluorescence microscopy as described previously (Rinne *et al.*, 2005), and photographed with a digital camera (Nikon Coolpix 995).

### RNA extraction and quantitative RT–PCR analysis

The apex and every second one of the subtended AXBs up to node 30 (counted from the apex) were collected from LD plants. In parallel, apices and maturing AXBs at the even nodal positions 2–14 were collected from SD plants at SD weeks 2, 3, and 5. Two types of decapitation experiments were carried out. In the first experiment, the five AXBs immediately under the cut were collected at 8 d post-decapitation. In the second experiment, the first AXB under the cut was collected after 1, 2, 3, 5, and 7 d. For each data point, RNA was extracted from six plants, and divided into two biological replicates, each containing material from three individual plants. To exclude possible diurnal variations in gene expression, sampling was carried out at exactly the same time of day for all analyses.

RNA was extracted from 0.2g of frozen tissue and ground in a mortar with 750 μl of extraction buffer (Qiagen RTL buffer, containing 1% PVP-40). After addition of a 0.4vol. of KoAC at pH 6.5 and further grinding, the solution was transferred to a 2ml tube, incubated on ice for 15min, and centrifuged at 12 000rpm at 4 ^o^C for 15min. The supernatant was transferred to a 1.5ml tube, and a 0.5vol. of 100% EtOH was added. The mix was transferred to two RNeasy-spin columns and further processed in accordance with instructions of the Qiagen Plant RNA isolation kit. RNA was DNase (Ambion) treated, cleaned using the total RNA purification system ‘Purelink RNA mini kit’ (Invitrogen), and reverse transcribed using SuperScriptIII reverse transcriptase (Invitrogen). Quantitative reverse transcription–PCR (qRT–PCR) analyses were performed with the ABI Prism 7500Fast sequence detection system using SYBR Green PCR master mix (Applied Biosystems). Transcript levels were normalized using an actin gene. Gene-specific primer sequences for the analyses were designed using Primer3 (http://frodo.wi.mit.edu/primer3) (Supplementary Table S1 at *JXB* online).

### Transformation and *in vitro* culture of hybrid aspen

For vector construction and *Agrobacterium*-mediated transformation, genomic clones of T89 *GH17_44* and *GH17_102* were amplified and subsequently cloned into the pMDC32 destination vector (Curtis and Grossniklaus, 2003) using the Gateway system (Invitrogen), replacing the *ccdB* gene downstream of the dual *Caulifower mosaic virus* (*CaMV*) *35S* promoter. The overexpression vectors were transformed into the *Agrobacterium tumefaciens* strain GV3101 (pMP90). Hybrid aspen (clone T89) was first grown *in vitro* under sterile conditions for 4–5 weeks (photoperiod 18h, light intensity 28 µmol m^−2^ s^−1^, temperature 20 ^o^C). Explants of these plants were used for *Agrobacterium*-mediated transformation (Häggman *et al.*, 2003). Briefly, 3–5mm long internodal stem segments were cut and placed on solid callus production medium, hereafter referred to as MS1 [half-strength Murashige and Skoog (1/2× MS) medium; Duchefa, M0222], which contained 2% sucrose, 0.5 µM 6-benzylaminopurine (BAP; Sigma B3408), 4 µM 2,4-dichlorophenoxyacetic acid (2,4-D; Fluka, 31518), and 0.7% agar (pH 5.6). Stem segments were incubated under light for 3 d prior to co-cultivation with *Agrobacterium*. Fresh cultures of *A. tumefaciens* strain GV3101, containing the binary plasmids *Pro35S::GH17_44* or *Pro35S::GH17_102*, were grown in Luria broth (LB) medium (1.0% tryptone, 0.5% yeast extract, and 1.0% NaCl) containing antibiotics (20 µg ml^−1^ rifampicin, Sigma, R3501), 30 µg ml^−1^ gentamicin (Sigma, G6896), and 100 µg ml^−1^ kanamycin (Sigma, K4378). The cultures were grown until OD_600_ (optical density) of ~0.5. Subsequently, the cultures were centrifuged for 10min. at 3000rpm, washed once in distilled water, and re-suspended in a MS1 solution which was supplemented with 2% sucrose to an OD_600_ of ~0.5. Acetosyringone (Sigma, D134406) was added to the culture in a final concentration of 20 µM, and cultures were further grown at room temperature for 1h with shaking (60rpm). The explants were co-cultured with pre-incubated *A. tumefaciens* cells for 4h (room temperature, 60rpm), and then incubated on MS1 plates for 48h in the dark. Thereafter *Agrobacterium* cells were removed by rinsing the explants three times in 1/2× MS liquid medium containing 2% sucrose, and twice in 1/2× MS liquid medium containing 2% sucrose, 300mg l^−1^ vancomycin (Duchefa, V0155), and 500mg l^−1^ claforan (cefotaxime sodium, Duchefa, C0111), for 15min per wash (room temperature, 60rpm). The explants were blotted on a sterile filter paper and transferred to MS1 plates with antibiotic selections [15 µg ml^−1^ hygromycin (Sigma, H9773) and 250 µg ml^−1^ claforan] to initiate callus growth. At a size of ~5mm, the calluses were transferred to the shoot regeneration medium MS2 [1/2× MS medium containing 2% sucrose, 0.1 µM thidiazuron (TDZ; Duchefa, T0916), and 0.7% agar at pH 5.6], with antibiotic selections (15 µg ml^−1^ hygromycin and 250 µg ml^−1^ claforan). Approximately 5cm tall plantlets were transferred to the rooting medium MS3 [1/2× MS medium supplemented with 100mg l^−1^ myo-inositol, 2.85 µM indole acetic acid (IAA; Sigma, I2886), and 0.8% agar at pH 5.6] without any antibiotic selection. Rooted cuttings were transferred to soil for greenhouse growing. Expression of overexpressed genes in different lines was analyzed by qPCR in leaves, stems, and AXBs. The number, position, and length of sylleptic branches was monitored after the plants had grown in soil in the greenhouse for 2 months.

### Bioinformatics

BLAST searches in GenBank and the *P. trichocarpa* genome v2.0 (Tuskan *et al.*, 2006) databases (http://www.ncbi.nlm.nih.gov/BLAST; http://www.phytozome.net) was used to identification of GH17 and GA biosynthesis and signaling genes. ClustalW (http://www.ebi.ac.uk/Tools/msa/clustalw2) was used to perform multiple sequence alignments. The PLACE database was used to compare plant *cis*-acting regulatory DNA elements in the putative promoter region (1000 bp upstream) of *GH17* genes of *P. trichocarpa* (http://togodb.biosciencedbc.jp/togodb/view/place_main) (Higo *et al.*, 1999). The expression map of Arabidopsis genes, e-FP browser, was used to search the expression patterns of orthologous genes in the GH17-family (http://bar.utoronto.ca/efp/cgi-bin/efpWeb.cgi). Gene-specific primer sequences for the qPCR analysis were designed using Primer3 (http://frodo.wi.mit.edu/primer3).

## Results

### Time frame of axillary and terminal bud formation

**Fig. 1.**
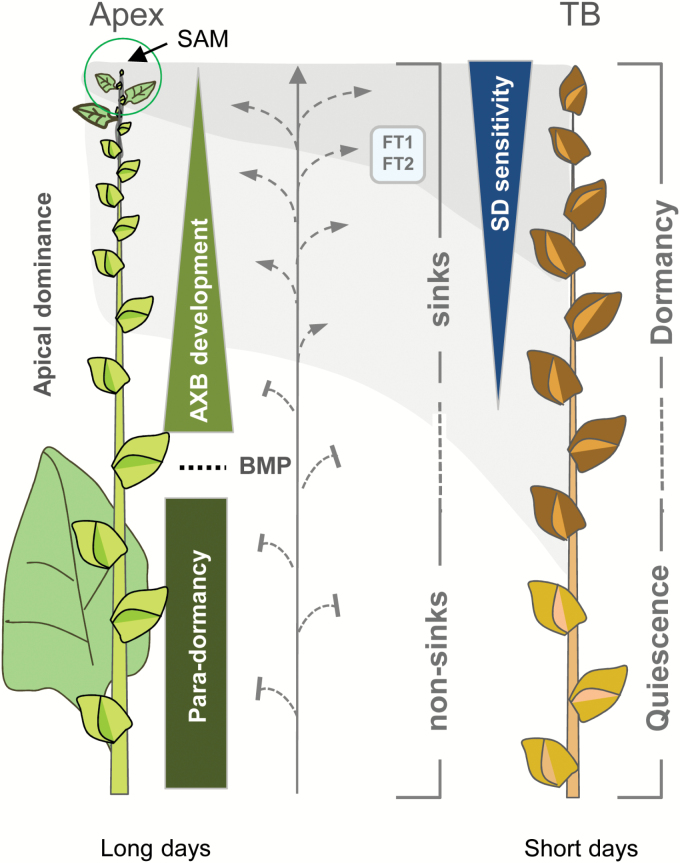
Conceptual scheme for comparative analysis of para-dormancy and dormancy. Under long day conditions (left), the shoot apical meristem (SAM) produces axillary meristems that give rise to axillary buds (AXBs), which gradually enlarge (upright triangle) until they reach their final size at the bud maturation point (BMP, stippled line). At the BMP, the full-grown AXB contains a complete dwarfed embryonic shoot, which is maintained throughout para-dormancy (rectangle). Under short days (right), all developing buds can establish dormancy. This includes the emerging terminal bud, the AXMs that were still in the apex when SDs were applied (dark shadow), as well as the young AXBs that were well above the BMP (light shadow). ‘SD-sensitivity’, reflecting signal import under short days (blue box, FT1/FT2), is indicated by an inverted triangle (dark blue). The number of AXBs is arbitrary (adapted from Rinne *et al.*, 2015).

AXB formation in hybrid aspen is a default process (Fig. 1). Branching of the T89 clone is proleptic, meaning that under normal growth conditions AXBs only give rise to branches after a period of (winter) dormancy. This makes it an excellent system to investigate AXB development, para-dormancy, and decapitation-induced branching, as spontaneous bud burst is absent. AXBs contain a dwarfed shoot system, the development of which is completed at the BMP (Fig. 1). In our experimental conditions, reaching this point takes ~4 weeks (Rinne *et al.*, 2015). By then, the AXB contains ~10–12 embryonic leaves. In comparison, TB formation is a non-default process that is under strict control by SDs, and results in dormancy establishment in ~5 weeks. To enable comparison of AXBs and TBs at a similar developmental stage, we first aimed to establish the point in time at which the SAM transitions to a TB. Because molecular markers for scale initiation, an early sign of the transition, have not been identified, an indirect growth analysis method was used (Materials and methods). The analysis showed that under SDs two leaves (±0.9) were produced before leaf production ceased and TB formation started. Under the assumption that the plastochron did not change, the reprogramming of leaf primordia to scale primordia took 3 d. In an alternative procedure, plants were exposed to SDs for a restricted number of days before returning them to LDs, to assess the earliest time point at which the apex would become morphologically affected. A very short exposure to SDs, for 2–4 d, did not produce visible signs of TB formation, but newly formed internodes could be slightly shorter, reducing the overall height of the plant (Supplementary Fig. S1). After an exposure of 6–12 d, plants sporadically formed sylleptic branches, suggesting that during SD exposure apical dominance was weakened by the tendency to form a TB. After a longer SD exposure of 14–21 d, non-reversible scale-like stipules formed although the leaf lamina expanded. An exposure of 21 SDs or longer seriously compromised reversion to normal growth (Supplementary Fig. S1). Assuming that diminished apical elongation and weakening of apical dominance directly preceded TB initiation (i.e. changes in the SAM and scale initiation), the results indicate that the SD response at the SAM was as early as 3 d after the start of SD exposure. The time frame to form a dormant TB at week 5 is thus roughly similar to the 4 weeks needed to form a completed AXB.

### Axillary buds amass lipid bodies in long and short days

**Fig. 2.**
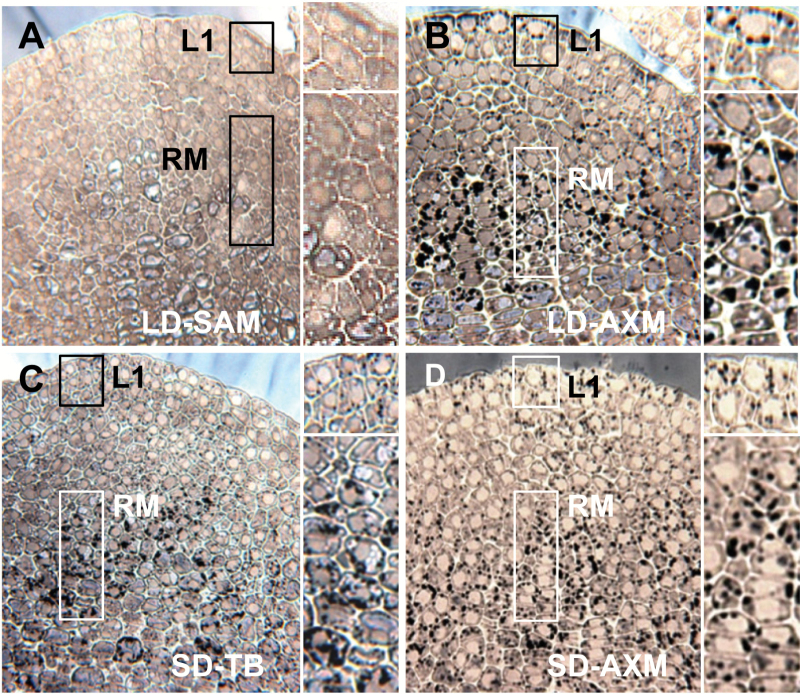
Lipid bodies in apical and axillary meristems. Median longitudinal sections of shoot apical meristems (SAMs) (A and C), and axillary meristems (AXMs) of axillary buds (B and D) of long day (LD) plants (A and B) and plants after 4 weeks of short days (SDs) (C and D). Samples were stained with Sudan II (black) for lipid. The LD SAM has very few, light blue-staining lipid bodies (A), whereas all other SAM states possess prominent black lipid bodies (B–D). Boxed areas (A–D) of the uppermost meristem layer (L1) and the rib meristem (RM) are enlarged on the right. TB, terminal bud.

Structural analyses previously showed that the developmental trajectories of TBs and AXBs converge on a shared morphogenetic program (Rinne *et al.*, 2015). We here investigate if para-dormant and dormant AXBs also share the unique cellular features that characterize dormant TBs. Cyto-histological studies, using the lipid stain Sudan II (black), showed that the lipid bodies that amass in the SAM and rib meristem of SD-induced dormant TBs (van der Schoot *et al.*, 2011) are also prominent in para-dormant and dormant AXBs. A direct comparison showed that the amount of lipid bodies was very similar (Fig. 2). In all cases, intensely black lipid bodies crowded the cytoplasm of the SAM and the rib meristem cells. In contrast, the apex of growing LD plants contained very few lipid bodies that stained light-blueish (Fig. 2A). In para-dormant AXBs, lipid bodies were particularly prominent in the upper cell layer of the central zone (L1), and in the rib meristem (Fig. 2B). In conclusion, lipid body accumulation appears to be an integral part of bud development as such, preceding para-dormancy as well as dormancy. Another significant feature that is shared by all buds is the loss of cellular water. Under LDs, AXBs desiccated considerably, lowering their water content in developing AXBs from 80% to ~60% around the BMP, and then further during para-dormancy to 50% (Supplementary Fig. S2). Taken together, lipid body accumulation and desiccation accompany the formation of TBs as well as AXBs.

### Expression profiles of GA pathway genes in developing buds

**Fig. 3.**
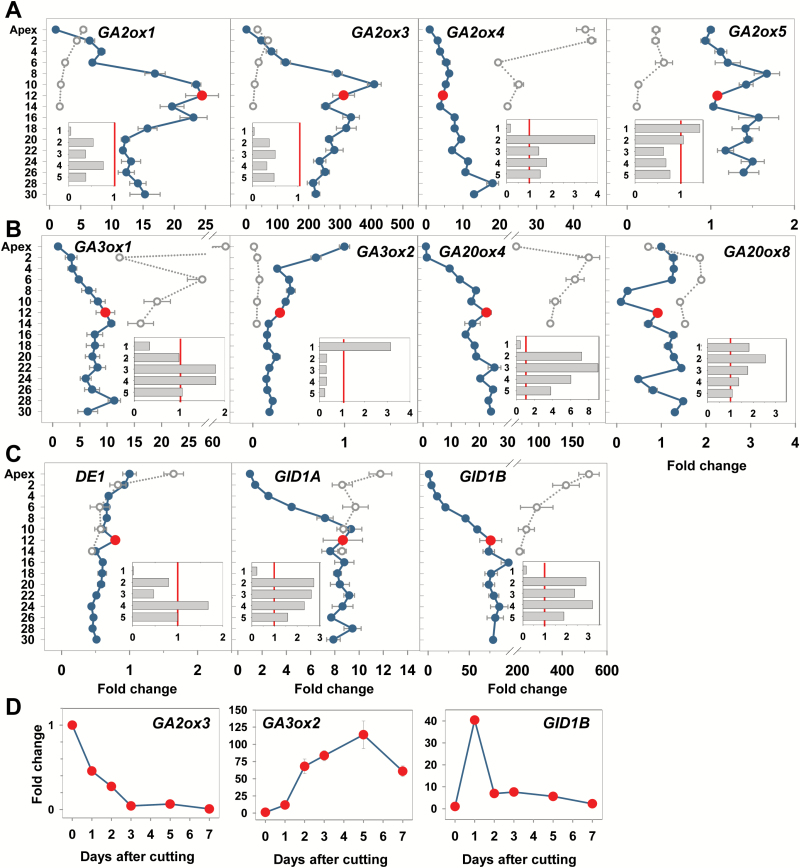
Expression analysis of selected gibberellin (GA) pathway genes during axillary bud (AXB) development under long and short days, and after stem decapitation. (A) GA-deactivating GA2-oxidase-like genes, *GA2ox1*, *GA2ox3*, *GA2ox4*, and *GA2ox5*. (B) GA biosynthesis genes, belonging to GA3-oxidase (*GA3ox1* and *GA3ox2*) and GA20-oxidase families (*GA20ox4* and *GA20ox8*). (C) DELLA-like gene (*DE1*) and two GID1-receptor-like genes (*GID1A* and *GID1B*). (A–C) Fold change under long days in the apex and AXBs (LD; blue dots and lines) and after 5 weeks of short days (SD; open circles, stippled lines) for the terminal bud and AXBs up to node 14. The red dot indicates the expression level in AXB 12 of intact plants, at the bud maturation point (BMP). After stem decapitation at the BMP, gene expression was measured at day 8 in five successive AXBs (1–5) directly proximal to the cut (A–C, insets) (*x*-axis fold change), and (D) during a 7 d period in AXB 12, proximal to the cut. The values (A–C) are calculated relative to the apex expression level (set at 1), and in the inset relative to each individual AXB position before decapitation (set at 1, red line) and (D) relative to the AXB position 12 before decapitation. Values represent means of six plants ±SE, analyzed in two pooled samples.

Xylem feeding of GA_4_ activates dormant AXBs and induces them to grow out (Rinne *et al.*, 2011), suggesting that the dwarfing of the embryonic shoot is caused by a deficiency in GA signaling. We investigated this possibility by analyzing the expression of genes that are central to GA catabolism, biosynthesis, and signaling in AXBs of different developmental stages and activity states. The results show that the GA pathway genes that function in dormancy cycling at the shoot apex have expression patterns characteristic of developing, para-dormant, and dormant AXBs (Fig. 3).

#### GA catabolism

The *GA2-OXIDASE*-family (*GA2ox*) of GA-catabolizing genes (Gou *et al.*, 2011) functions in reducing the levels of bioactive GA. Several *GA2ox* members were expressed in AXBs (Fig. 3A). The transcript levels typically increased in developing AXBs (i.e. until they reached the mature stage around the BMP). *GA2ox2* expression was below the detection limit (not shown), but *GA2ox1*, *GA2ox3*, and *GA2ox4* were significantly up-regulated during AXB development, from 10- to 400-fold. *GA2ox6* was very modestly up-regulated (not shown). *GA2ox1* and *GA2ox3* expression was very low in proliferating apices compared with developing AXBs, suggesting a comparatively diminished catabolism of GA in the apex (Fig. 3A). This is in agreement with the consensus view that GA is necessary to facilitate shoot elongation growth. Consistent with this, the SD-induced cessation of apical growth and TB formation resulted in the up-regulation of three of the four GA catabolism genes (Fig. 3A). *GA2ox4* stood out because it was strongly up-regulated not only in dormant TBs but also in the AXBs that were still developing when SD exposure started. Because these developing AXBs also become dormant (Rinne *et al.*, 2015), the GA catabolism gene *GA2ox4* might be central in dormancy establishment.

#### GA biosynthesis

The two selected members of the *GA3-OXIDASE*-family (*GA3ox*) that function in the last biosynthesis step of biologically active GA showed opposite expression patterns (Fig. 3B). *GA3ox1* positively reflected AXB development. It was hardly expressed in growing apices, gradually up-regulated in developing AXBs, and maintained at a relatively steady level below the BMP. In contrast, *GA3ox2* was characteristic of apical growth and elongation. It was highly expressed in the apex, but considerably down-regulated during AXB development (Fig. 3B). Consistent with these distinct patterns, SDs induced up-regulation of *GA3ox1* and down-regulation of *GA3ox2* in TBs and developing AXBs. The members of the *GA20-OXIDASE*-family (*GA20ox*), which produce precursors for the *GA3ox*-family, were also differentially regulated. *GA20ox8* showed very little change during AXB development and under SDs. As this gene is chilling regulated (Rinne *et al.*, 2011), it might function predominantly in dormancy release. In contrast, *GA20ox4* (Fig. 3B) and *GA20ox3* (not shown) were up-regulated during AXB development under LDs, reaching a steady expression level around the BMP. *GA20ox4* was further up-regulated to a much higher level in developing TBs and AXBs that established dormancy under SDs (Fig. 3B), suggesting that during para-dormancy and dormancy high levels of biologically inactive precursors are produced. This would allow a rapid production of biologically active GA if needed, because only the final GA3ox enzyme has to be produced.

#### GA signaling

Expression of *DE1*, a *DELLA*-like gene, was low and stable in AXBs up to the BMP, and slightly down-regulated in older AXBs. Under SDs, it was exclusively up-regulated in TBs (Fig. 3C). Because DELLA proteins interact with a GA receptor, thereby affecting tissue sensitivity to GA, we also analyzed the expression of two putative homologs of the rice gene *GID1*, encoding the GID1 receptor (Ueguchi-Tanaka *et al.*, 2005). These genes, *GID1A* and *GID1B*, align with Arabidopsis *GID1A* and *GID1B*, respectively. Both genes were substantially up-regulated during AXB development, up to 10- and 100-fold, respectively, and further up-regulated during dormancy. This is congruent with the hypothesis that para-dormant and dormant buds are GA deficient as well as GA sensitized.

### Unique expression patterns of PD callose-related GH17 genes

**Fig. 4.**
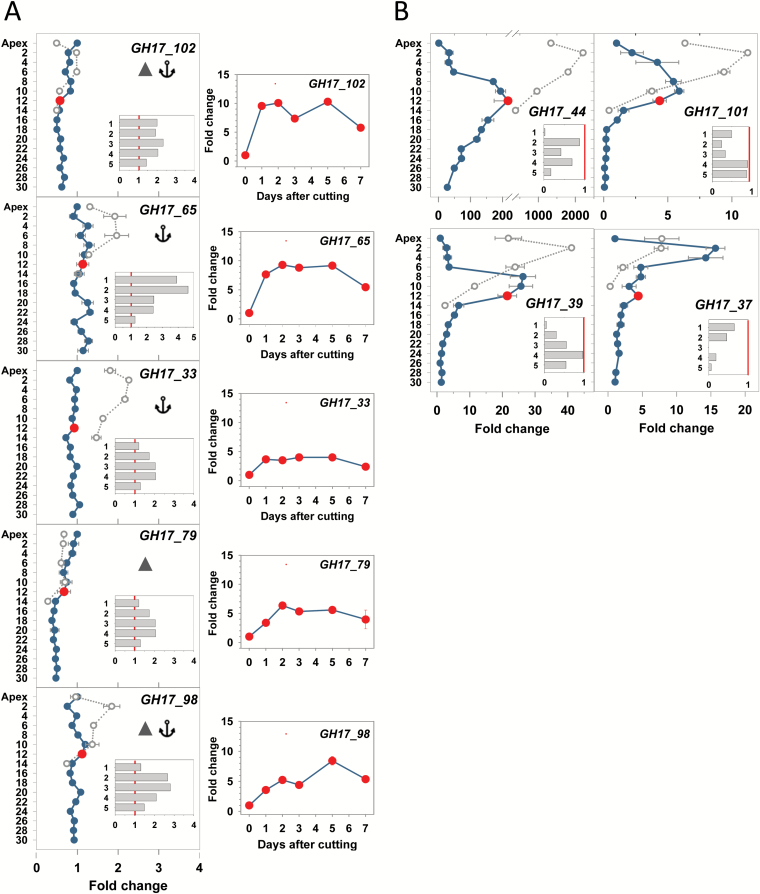
Expression analysis of α-clade and γ-clade GH17 genes in the apex and axillary buds (AXBs) developing under long and short days, and after stem decapitation. (A) α-Clade genes with a CBM43 domain (solid triangle) or GPI anchor (anchor), and (B) γ-clade genes with putative lipid body localization. Gene names and protein domains are indicated in each individual panel. Fold change under long days in the apex and AXBs (LD; blue dots and lines) and after 5 weeks of short days (SD; open circles, stippled lines) for the terminal bud and AXBs up to node 14 (A, left column; B). The red dot indicates the expression level in AXB 12 of intact plants, at the bud maturation point (BMP). After stem decapitation at the BMP, gene expression was measured at day 8 in five successive AXBs (1–5) directly proximal to the cut (insets; *x*-axis fold change), and (A, right column) during a 7 d period in AXB 12, proximal to the cut. The values are calculated relative to the apex expression level (set at 1), and in the inset relative to each individual AXB position before decapitation (set at 1, red line) and (A, right column) relative to AXB position 12 before decapitation. Values represent means of six plants ±SE, analyzed in two pooled samples.

The involvement of GA-regulated, PD-targeting, and callose-hydrolyzing members of the 1,3-β-glucanase-family (GH17) was previously investigated in relation to TB dormancy cycling (Rinne *et al.*, 2011). We here analyzed their comparative expression during AXB development, para-dormancy, and dormancy. The α-clade members (Fig. 4A) are post-transcriptionally modified for excretion to the cell wall. They possess a GPI anchor (GH17_102, GH17_65, and GH17_33) and/or a CBM43 (GH17_102, GH17_79, and GH17_98). The CBM43 module facilitates their targeting to cell wall callose that is present around PD orifices (Doxey *et al.*, 2007; Levy and Epel, 2009; Simpson *et al.*, 2009). Under LDs, expression of the α-clade genes *GH17_102* and *GH17_79* gradually declined from apices toward the AXB at the BMP, with apices having a twice as high expression level (detailed in Supplementary Fig. S3). In contrast, expression of *GH17_65*, *GH17_33*, and *GH17_98* was more equal in the apex and all subtending AXBs (Fig. 4A). Under SDs, *GH17_102* and *GH17_79* were also down-regulated in the dormant TB. In contrast, *GH17_65* and *GH17_33* were up-regulated in dormant AXBs and TBs, while *GH17_98* was only up-regulated in dormant AXBs (Fig. 4A). Regulation of the γ-clade genes (*GH17_44*, *GH17_101*, *GH17_39*, and *GH17_37*) was distinctly different from that of the α-clade (Fig. 4B). Their expression levels increased strongly but transiently during AXB development up to the BMP, closely correlating with the accumulation of lipid bodies, where they might be stored for later recruitment (Paul *et al.*, 2014*b*). In the dormant TBs and AXBs, all four γ-clade genes were substantially up-regulated, although less so in the case of *GH17_37* (Fig. 4B).

### Decapitation induces GA biosynthesis and GH17 genes, and activates AXBs

Although in the T89 clone AXBs do not branch out in the year in which they are formed, they can be induced to do so by decapitation. Branching depends on functional symplasmic connections between the stem and the AXB, which is expected to require both GA and GA-regulated GH17 members that diminish callose at PD and sieve plate pores (Levy and Epel, 2009; Rinne *et al.*, 2011). We therefore analyzed the expression of GA pathway genes in conjunction with the GA-induced GH17 members. Para-dormant AXBs possessed considerable amounts of callose at PD, especially in the SAM, provascular tissue, and sieve tubes plus surrounding ground tissue of the embryonic stem, a wedge-like extension that connects the SAM to the main stem (Supplementary Fig. S4A–C). Activated AXBs of single-node cuttings showed that in 3 d callose deposits were strongly diminished (Supplementary Fig. S4D), before signs of bud burst occurred (Rinne *et al.*, 2015). For gene expression analyses, plants were decapitated directly above the BMP, at nodal position 12, and gene expression was analysed after 8 d in five successive AXBs proximal to the cut (insets of Figs 3 and 4). Although all five AXBs might become activated, those closest to the cut will usually win the competition and become the new leading shoot (Rinne *et al.*, 2015). Therefore, the most proximal AXB was chosen to analyze the expression of a number of selected genes in more detail. The data show that significant changes in gene expression took place within 24h. In all cases, decapitation substantially affected the expression of GA catabolism, biosynthesis, and signaling genes (Fig. 3).

The GA-catabolizing *GA2ox* genes (*GA2ox1* and *GA2ox3*), which were significantly up-regulated during AXB development, were down-regulated 1 week after decapitation in all five proximal AXBs, but most significantly in the most proximal one (Fig. 3A, insets). The GA-catabolizing enzyme, *GA2ox4*, which was somewhat up-regulated during AXB development and more substantially during dormancy establishment, was reduced only in the AXB closest to the cut (Fig. 3, inset). The GA-catabolizing gene *GA2ox5* did not show a decapitation-induced down-regulation in the proximal AXB, but was up-regulated instead (Fig. 3A, insets). A time-resolved analysis of one member in this family, *GA2ox3*, showed that in the uppermost AXB, the gene was down-regulated within a single day to the low levels characteristic of a proliferating apex (Fig. 3D).

The GA biosynthesis gene families *GA3ox* and *GA20ox* were both affected by decapitation. The genes *GA3ox1* and *GA20ox4*, which were up-regulated during AXB development, were down-regulated in the proximal AXB. In contrast, the four lower AXBs, which commonly did not produce branches, up-regulated the expression (Fig. 3B, insets). In sharp contrast to *GA3ox1* and *GA20ox4*, the gene *GA3ox2* was up-regulated in the uppermost AXB to ~10- and 70-fold during the first and second day post-decapitation, respectively (Fig. 3D). By day 5, it reached a 100-fold expression level, which subsequently declined to the level typical of growing apices. In the remaining four AXBs, the expression of *GA3ox2* was lower at 8 d post-decapitation (Fig. 3B, inset). Notably, this gene is characteristically expressed in the growing apex of non-decapitated plants, while it is repressed in dormancy (Fig. 3B). Thus, *GA3ox2* appears to be crucial in activation of the proximal AXB and may enable it to become the new leading shoot.

GA signaling was also severely affected by decapitation. At 8 d post-decapitation, expression of the *DELLA1*-like gene *DE1* was strongly reduced in the three uppermost AXBs (Fig. 3C, inset). The GA receptor genes *GID1A* and *GID1B*, which were highly expressed at the BMP in intact plants, were considerably down-regulated in the AXB proximal to the cut 8 d post-decapitation (Fig. 3C, inset). However, a day-by-day analysis of *GID1B* showed that in this AXB the expression shortly peaked at the second day after decapitation (Fig. 3D). The *GID1A* and *GID1B* repression occurred when the AXB had become more elongate and the GA biosynthesis had increased. In contrast, in the lower AXBs, *GID1A* and *GID1B* were up-regulated 2- to 3-fold, in agreement with the low expression of the GA biosynthesis gene *GA3ox2* (Fig. 3A, B, insets). The expression ratio of *GID1A* and *GID1B* to *GA3ox2* was also high during AXB and TB development, reflecting their inhibited state.

As anticipated, decapitation also affected the GA-regulated genes that encode the PD-related GH17-family proteins. The expression of growth- and GA_4_-regulated #x03B1;-clade GH17 genes (*GH17_102*, *GH17_65*, *GH17_33*, *GH17_79*, and *GH17_98*) was up-regulated during the first day post-decapitation, up to 10-fold in the case of *GH17_102*, and remained high during AXB activation (Fig. 4A). The γ-clade GH17 genes *GH17_39*, *GH17_101*, *GH17_37*, and *GH17_44* that were strongly up-regulated during AXB development were down-regulated by decapitation, especially in the proximal AXB (Fig. 4B, insets).

### Overexpression of GH17 members in hybrid aspen induces distinct branching phenotypes

**Fig. 5.**
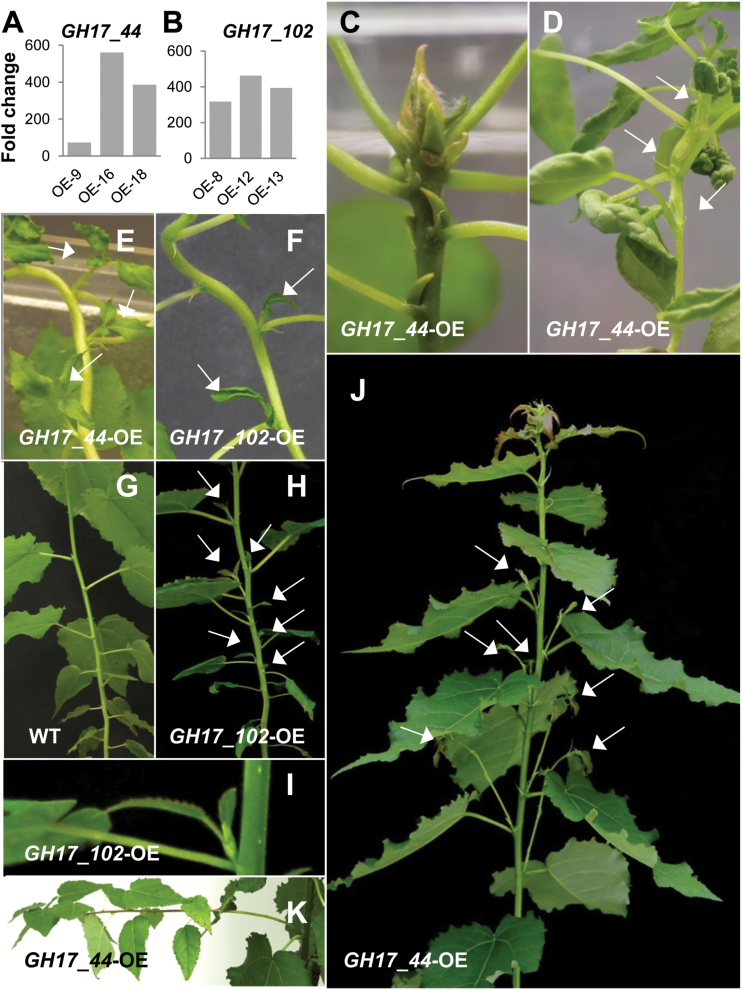
Phenotypes of young hybrid aspen lines overexpressing PD-associated *GH17*-family genes. Expression levels of (A) *GH17_44* and (B) *GH17_102* of three independent lines, compared with the wild type (WT=1). Phenotypes of *GH17_44* lines in (C–E) tissue culture and (K) after growth in soil. Phenotypes of *GH17_102* lines in (F, H) tissue culture and (I) after growth in soil. (G) Control wild-type plant for *GH17_102*-OE in soil. (C) Detail of TB, developing spontaneously in *GH17_44* lines. (D) In the most severe lines, meristem function was compromised, resulting in fasciation, consumption, and sympodial branches (arrows). (E, F) Spontaneous AXB branching affecting a number of subsequent buds (arrows). (H) AXBs in *GH17_102* lines were typically activated in young plants at the lower part of the stem. These ‘branches’ (arrows) remained small, and typically produced only a few leaves (I), while AXBs in *GH17_44* lines produced long and thin branches (J, K). For details of three *GH17_44*-OE lines as well as three *GH17_102*-OE lines see Supplementary Table S2.

The distinct expression patterns of the α-clade and γ-clade GH17 members during apical growth, AXB development, AXB para-dormancy, and decapitation-induced AXB activation warrant the hypothesis that these enzymes have unique roles in developmental processes. To probe their putative function, we constitutively overexpressed a representative of each clade in the T89 clone of hybrid aspen (Fig. 5A, B). *GH17_102* was selected from the α-clade because its expression was highest in the apex (Fig. 4A) and it was closely co-expressed with *PIN1* and the meristem-related gene *WUS* (Rinne *et al.*, 2015). From the γ-clade we selected *GH17_44*, encoding a lipid body-related protein, because it was virtually absent from the proliferating apex, but significantly up-regulated in AXB (Fig. 4B). Other reasons for selecting these genes relate to the differences in their promoter regions.

Promoter analysis, using a publicly available database of plant *cis*-acting regulatory DNA elements http://togodb.biosciencedbc.jp/togodb/view/place_main, showed that the promoter region (1000bp upstream) of *GH17_102* was enriched with multiple central elements that imply auxin and GA regulation (Supplementary Table S3). *GH17_102* was the only one among the studied *GH17*-family genes which has the UP2 motif in its promoter region. In Arabidopsis, this motif relates to decapitation-induced AXB activation (Tatematsu *et al.*, 2005). In addition, *GH17_102* possesses the same target sequences for the meristem-specific transcription factors LEAFY and WUS as are found in the intron of the *AGAMOUS* gene (Lohmann *et al.*, 2001). In contrast, lipid body-related *GH17* genes possess the sugar-responsive *cis*-element SRE in their promoter region (Supplementary Table S3), which in Arabidopsis is also present in a number of genes that are down-regulated by decapitation (Tatematsu *et al.*, 2005).

**Fig. 6.**
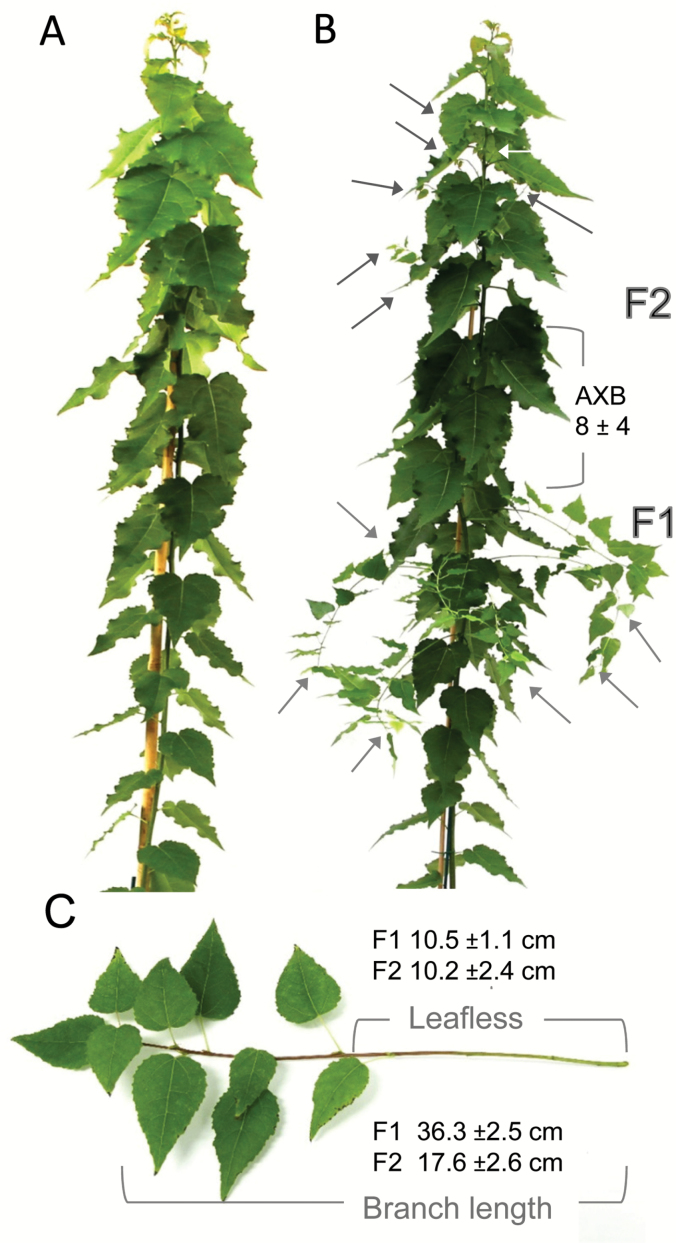
Recurrent AXB activation in hybrid aspen overexpressing the lipid body-related *GH17_44* gene. (A) The wild-type plant lacks branches. (B) *GH17_44* overexpressors (OE-9) spontaneously flushed, typically producing branches from ~7 (±2) successive AXBs. Arrows mark the first and the second flush (F1 and F2) branches, separated by 8 (±4) inhibited AXBs (OE-9). (C) The branches remained thin and showed characteristics typical of a sylleptic branching style, with a long leafless basal part. The average branch length and the average length of the leafless part are indicated (±SE). For details of three *GH17_44*-OE lines as well as three *GH17_102*-OE lines see Supplementary Table S2.

Since *GH17_102* was predominantly expressed in proliferating meristems, we anticipated that its overexpression would release the repressed para-dormant state of AXBs, thereby changing the proleptic branching habit of the T89 clone to a sylleptic one (Figs 5G, 6A; Supplementary Table S2). Consistent with this, *GH17_102* overexpression lines (Fig. 5B) showed AXB burst and formation of sylleptic branches (Fig. 5F, H). Notably, AXB branching was not sporadic but patterned. Branches gradually emerged in an acropetal pattern, suggesting that once the AXBs had reached a certain level of development and maturation they were able to burst and produce a branch. We observed this in tissue culture plants and in younger soil-grown plants (Fig. 5F, H). When plants grew taller than 0.5 m this acropetal branching pattern diminished. All lines were SD responsive and made TBs in the same time frame as the wild type.

Because *GH17_44* transcripts were virtually absent from the proliferating apex, we expected that its overexpression would interfere with apex function. Indeed, all overexpressor lines had apical deviations. Two of the *GH17_44* overexpression lines (lines 1 and 2) produced large terminal buds and pronounced AXBs under LDs, and as early as the tissue culture stage (Fig. 5C). Although the large AXBs could burst after decapitation, the apical meristems of the emerging branches would again become arrested. Other, more severely affected *GH17_44* overexpression lines showed fasciation and SAM termination, whereas some lines continued growth repetitively from the uppermost AXB in a sympodial manner (Fig. 5D). At least eight of the *GH17_44* overexpression lines (lines 8–12 and 16–18), those with less enhanced expression levels, were able to grow in soil. Contrary to expectation, these lines also produced branches (Figs 5J, 6), not from mature AXBs like the *GH17_102* overexpression lines but from numerically younger AXBs. Remarkably, branching occurred in distinct recurrent flushes, even when plants became older (Supplementary Table S2). Line 9 displayed the most severe branching phenotype (Fig. 6B), reminiscent of the behavior of wild-type plants that were exposed for 6–12 d to SD conditions, and often branched from the uppermost AXB (Supplementary Fig. S1). As overexpression of *GH17_44* could induce spontaneous bud formation in some lines, it seems likely that recurrent branching in less severe *GH17_44* lines was due to the recurrent formation of a TB-like structure, which then causes syllepsis indirectly.

## Discussion

**Fig. 7.**
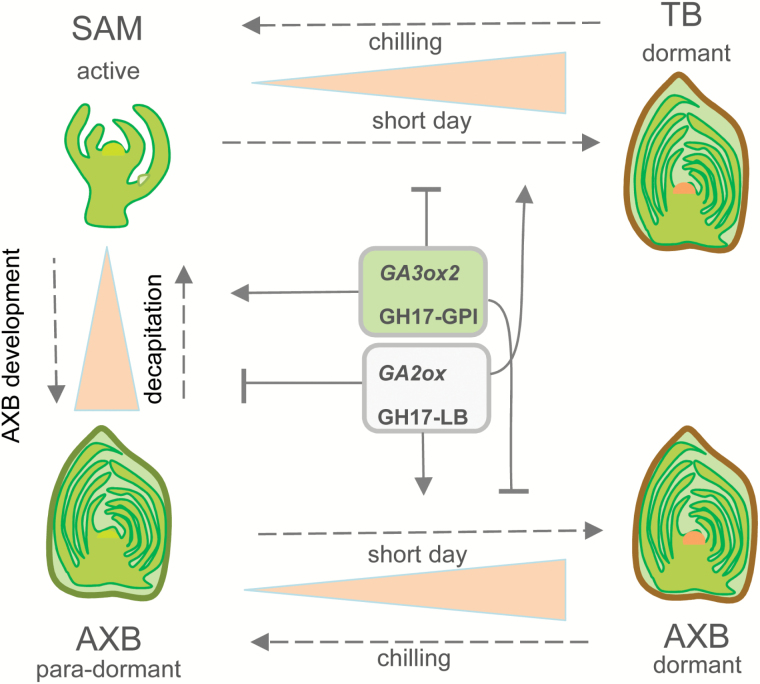
Schematic model of GA- and GH17-based mechanisms that facilitate identity swapping of SAM and AXMs. The SAM produces AXBs that gradually develop (arrow), and become para-dormant. Decapitation activates the para-dormant AXBs, the axillary meristem which becomes the SAM of the side shoot. This requires up-regulation of *GA3ox2* and the GA-inducible GPI-anchored GH17 enzymes (green box) that remove callose at PD and sieve plate pores, while genes that function during AXB development, such as the GA-deactivating gene *GA2ox* and genes encoding the lipid body-associating GH17 enzymes are down-regulated (light gray box). Opposite regulation takes place in TBs and AXBs that develop wholly or partially under SDs and then establish dormancy. During chilling and subsequent bud burst, these processes are reversed. Stippled arrows refer to treatments; solid arrows and T-shaped lines refer to up-regulated and down-regulated genes, respectively. Pink triangles indicate relative changes in PD callose levels during development and when exposed to a short photoperiod and chilling.

The ‘architectural model’ of a tree species describes its branch geometry and patterns of reiterated development (Hallé *et al.*, 1978). However, the shape of a crown is developmentally plastic, and influenced by internal competition between branches as well as interactions with the environment (Tomlinson, 1983; Turnbull, 2005; Barthélémy and Caraglio, 2007; Costes *et al.*, 2014; Costes and Gion, 2015). How the crown maintains its operational hierarchy over multiple seasons is virtually unknown. In a recent study, we compared AXB and TB development as well as the expression of meristem-specific and branching-related genes, including *BRC1* and *MAX1* (Rinne *et al.*, 2015). This comparison showed that in terms of structure, development, and gene expression patterns, AXBs and TBs are very much alike, despite distinct differences in how and where they are formed. Furthermore, AXBs appeared morphogenetically active up to the BMP, where they become para-dormant, a phase where morphogenesis has simply ceased (Rinne *et al.*, 2015). The present results support a model in which GA pathway genes, and genes encoding GA-inducible 1,3-β-glucanases that degrade PD callose during dormancy release, are subject to a comparable regulation in development and activation of para-dormant AXBs (Fig. 7).

### Developing AXBs are sinks that accumulate lipid bodies

Dormant TBs of deciduous perennials can contain substantial amounts of lipid bodies. Known examples include birch (Rinne *et al.*, 2001) and hybrid aspen (Rinne *et al.*, 2011; van der Schoot *et al.*, 2011), and the evergreen Rhododendron (Lynch and Rivera, 1981). That dormant AXBs also possess lipid bodies is therefore not surprising (Fig. 2D). However, the finding that AXBs that develop under LDs also contain them (Fig. 2B) shows that lipid bodies are not exclusive indicators of dormancy, but rather an integral part of bud development. That such carbon reserves accumulate in young developing AXBs reflects that they are sinks that import from the phloem. Commonly, AXBs are not regarded as sinks; however, in hybrid aspen developing AXBs are not very different from the proliferating apex, except for the fact that cell enlargement and shoot elongation are postponed to the next season. That only AXBs above the BMP become dormant might relate to the fact that developing hybrid aspen buds are sinks, which are presumably accessible to photoperiodically regulated signals (Fig. 1), such as FLOWERING LOCUS T (FT) (Böhlenius *et al.*, 2006).

Lipid bodies are known to be abundant in seeds, but they are constituents of most plant cells, although in vegetative cells they are small and few in number (van der Schoot *et al.*, 2014). This changes during bud development when lipid bodies amass in quantities resembling those in seeds (Fig. 2; Rinne *et al.*, 2011; van der Schoot *et al.*, 2011). Like in seeds, which can also overwinter, lipid bodies in AXBs and TBs may serve as energy stores and confer cryo-protection during dehydration (Shimada *et al.*, 2008). Although less severe than in seeds, developing TBs and AXBs also dehydrate during development, irrespective of whether or not they establish dormancy (Faust *et al.*, 1991; Welling *et al.*, 1997; Supplementary Fig. S2). 1,3-β-Glucanases of the γ-clade can localize at lipid bodies, which target the plasma membrane and PD, as shown with immunolabeling and by eGFP tagging of 1,3-β-glucanases and the lipid body marker protein oleosin (Rinne *et al.*, 2001, 2011; Paul *et al.*, 2014*b*). The accumulated lipid bodies may function during chilling-induced release from dormancy as vehicles that shuttle 1,3-β-glucanases to PD to remove callose and restore the symplasmic organization of the SAM (Rinne *et al.*, 2011; Paul *et al.*, 2014*b*). The fact that lipid bodies and 1,3-β-glucanases of the γ-clade GH17-family also accumulate in developing AXBs suggests that they have a similar role as in TBs, which makes sense because AXBs as a rule overwinter in proleptic hybrid aspen before they can branch. Considering that lipid bodies are rapidly mobilized by xylem feeding of GA (Rinne *et al.*, 2011), it seems possible that the γ-clade GH17 members stored at lipid bodies may also have a role in activation of para-dormant AXBs, while subsequent outgrowth relies on GA_4_-induced 1,3-β-glucanases of the GH17 α-clade.

### GA pathway genes in AXB inhibition and activation

The present findings that AXBs do not express the growth-related GA biosynthesis gene *GA3ox2*, unless decapitated (Fig. 3B, D), while they do express the GA precursor genes (Fig. 3B), suggests that AXBs can rapidly produce biologically active GA by simply activating the last biosynthesis step. The implication is that para-dormant AXBs are poised for activation and outgrowth (Fig. 7). It is unclear how in para-dormant AXBs *GA3ox2* expression is maintained at a low level, but it could relate to a lack of auxin. Various GA biosynthesis genes are known to be induced by auxin (Figerio *et al.*, 2006), including a putative *Pisum* ortholog of the poplar gene *GA3ox2* (Ross *et al.*, 2000). While decapitation of hybrid aspen up-regulated the proximal AXB *GA3ox2* (Fig. 3D) and *PIN1*-like genes (Rinne *et al.*, 2015), the catabolism gene, *GA2ox3,* was down-regulated (Fig. 3A). Because GA catabolism appears to be restricted to a narrow zone under the SAM, protecting the SAM from the disturbing influence of GA (Sakamoto *et al.*, 2001; Jasinski *et al.*, 2005), this could direct GA-induced cell elongation to the rib zone. Considering that GA increases and stabilizes PIN1 abundance (Willige *et al.*, 2011), GA might stimulate auxin transport from the AXM to the rib zone, where it could promote its own auxin-mediated biosynthesis.

An additional crucial component in GA signaling is the GA receptor GID1 (Ueguchi-Tanaka *et al.*, 2005), the levels of which reflect GA sensitivity, as demonstrated in rice through overexpression of *GID1* (Ueguchi-Tanaka *et al.*, 2005). The substantial up-regulation of two *Populus* homologs of *GID1* during AXB development (Fig. 3C) may thus enhance the sensitivity of the system to GA, while it is already poised for activation by high expression of GA precursor genes. The data indicate that AXBs in general are considerably more sensitive to GA than the growing apex, as *GID1* expression levels were ~10- (*GID1A*) and 70- (*GID1B*) fold higher in LDs, and >200-fold higher in SDs (*GID1B*) (Fig. 3C, D). Thus, para-dormant AXBs might be poised for activation by tight regulation of GA pathway genes (Fig. 3B, C), as well as by the GA-responsive and rib meristem-resident gene *CENTRORADIALISLIKE1* (*CENL1*) (Rinne *et al.*, 2015). This poised state is balanced by high expression of GA-deactivating genes (Fig. 3A) and the branch inhibitor genes *BRC1* and *MAX1* (Rinne *et al.*, 2015).

In theory, in a GA-deficient but highly GA-sensitized system, a slight increase in GA biosynthesis could bring about AXB activation and outgrowth. The assumption that the AXBs represent such a system is supported by the finding that xylem feeding of the growth-related GA_4_ induces canonical bud burst, even when the AXBs are dormant (Rinne *et al.*, 2011). On the other hand, GA_3_, which is synthesized via a parallel pathway from the branch point GA_12_ precursor (Hedden and Thomas, 2012) and which is involved in chilling-induced release from dormancy, did not promote burst of dormant buds (Rinne *et al.*, 2011). The reason for this difference remains unclear, but could relate to different binding affinities of GA_4_ and GA_3_ for the GID1 receptor (Ueguchi-Tanaka *et al.*, 2005), or to the fact that they regulate different genes/paralogs. For example, in hybrid aspen, GA_3_ up-regulates genes encoding the bud development-related γ-clade members, while GA_4_ up-regulates growth-related α-clade members of the GH17-family (Rinne *et al.*, 2001, 2011). Together, this might explain why the capacity of applied GA to replace vernalization or chilling in different species has been inconsistent (Mutasa-Göttgens and Hedden, 2009).

The present conclusion that GA signaling has a pivotal role in regulating AXB activation is seemingly contradicted by the observation that GA biosynthesis mutants and plants that overexpress GA metabolism genes are often highly branched. Such observations, for example in *Pisum*, Arabidopsis, and turfgrass, seem to suggest that GA is not required for AXB outgrowth, and that GA may actually inhibit branching (Murfet and Reid, 1993; Silverstone *et al.*, 1997; Agharkar *et al.*, 2007; Lo *et al.*, 2008; Rameau *et al.*, 2015). Silencing of the GA catabolism gene *GA2ox* in tomato increased levels of GA_4_ in AXBs, but reduced branching (Martínez-Bello *et al.*, 2015). Considering the locally restricted expression of some *GA2ox* genes as discussed above, silencing may result in GA spilling over into the SAM. Silencing of *GA2ox* genes might then inhibit branching through experimentally induced mislocalisation of GA, compromising SAM integrity. In *Populus*, the reduction of GA levels by activation tagging of *GA2ox*, or the use of different promoters in overexpression studies, reduced or accelerated branching (Busov *et al.*, 2003; Mauriat *et al.*, 2011). The present suggestion that AXBs are GA deficient but GA sensitized could throw some light on these phenomena. In GA-deficient AXBs, overexpression of GA catabolism genes is unlikely to reduce GA levels much further, while it will affect the main shoot (see above; Willige *et al.*, 2011), decreasing the PATS and increasing branching due to reduced apical dominance.

### GA pathway genes in dormant AXBs

Judged from gene expression profiles, dormant AXBs may accumulate even more precursors of GA than para-dormant AXBs (Fig. 3B). Nonetheless, previous experiments with internode cuttings showed that this would be to no avail. As long as they do not receive sufficient chilling prior to decapitation, dormant AXBs fail to up-regulate *GA3ox2*, the gene that catalyzes the final GA biosynthesis step (Rinne *et al.*, 2011). Only after sufficient chilling at 5 ^o^C and subsequent return to 18 ^o^C is the gene up-regulated prior to bud burst (Rinne *et al.*, 2011). Together with the current data, this suggests that SDs repress *GA3ox2*, and that de-repression requires chilling followed by growth-promoting temperatures. This would be a mechanism that safeguards the AXBs from freezing damage, and ensures that growth is initiated at the proper time in spring (Rinne *et al.*, 2011). Coupling of photo- and thermo-periods in scheduling developmental events is also characteristic of other dormancy related genes, including *FT1* and *FT2* (Hsu *et al.*, 2006, 2011; Ruonala *et al.*, 2008; Rinne *et al.*, 2011; Brunner *et al.*, 2014). In all cases, these alterations might be expected to involve shifts in methylation and chromatin remodeling (Paul *et al.*, 2014*a*; Considine and Considine, 2016). Interestingly, in seeds of various species, light- and cold-inducible *GA3ox* genes are central to GA-regulated developmental events (Yamauchi *et al.*, 2004).

### The rib meristem as a target of branch regulators

A major function of GAs is to stimulate growth through cell elongation (Hedden and Thomas, 2012). This involves activation of the rib meristem and elongation of the descendent cells in the rib zone (Ruonala *et al.*, 2008). A role for *GA3ox2* in cell elongation is supported by the strong decapitation-induced up-regulation in the proximal AXB before it starts to telescope out (Fig. 3B). Interestingly, although reduced expression of the growth-related gene *GA3ox2* might be a major cause of dwarfing and hindrance to AXB outgrowth, expression of the non-growth-related paralog *GA3ox1* increased during AXB development (Fig. 3B). This suggests that in AXBs of hybrid aspen, *GA3ox1* and *GA3ox2* might have distinct expression domains as they function during bud formation and branching, respectively.

A hormone that opposes GA function as a branch regulator is strigolactone (Shinohara *et al.*, 2013). Although the roles of strigolactone in trees are as yet uncharted, a genuine strigolactone pathway has been identified in poplar (Czarnecki *et al.*, 2014). In hybrid aspen, *MAX1* genes are highly expressed in AXBs themselves, and down-regulated during decapitation, indicating that strigolactone could counteract GA locally in AXBs (Rinne *et al.*, 2015). For example, auxin-recruited GA might stimulate cell division and enlargement in the rib meristem and rib zone, as well as the establishment of an auxin export path to the main stem, while strigolactone opposes it by destabilizing PIN1 proteins (Willege *et al*., 2013). As in hybrid aspen AXBs develop while *MAX1* genes are up-regulated, it can be concluded that strigolactone does not impede cell division and morphogenesis, but it could contribute to inhibition of cell elongation. Such a role for strigolactone has been proposed for the dicots *Pisum* and Arabidopsis (Agusti *et al.*, 2011; de Saint-German *et al.*, 2013). On the other hand, in the monocot rice, strigolactone does not affect cell elongation but it negatively regulates cell division (Hu *et al.*, 2010). Based on the present data, a plausible scenario is that during AXB formation, GA_3_ can promote cell division because the simultaneous production of strigolactone and other inhibitors prevents elongation.

### AXB outgrowth requires high capacity delivery conduits

AXB activation and outgrowth can be thought of as conceptually distinct, but both probably depend on a functional stem–AXB connection. Activation of a para-dormant AXB triggers the re-initiation of morphogenesis, involving patterning processes at the AXM (SAM) and cell division activity in the rib meristem and rib zone. In the light of current understanding, this is regulated by cytokinin, auxin production in leaf primordia, and export to the stem (Müller and Leyser, 2011). Sugar is proposed to be the activation signal in caulescent *Pisum* (Mason *et al.*, 2014). In hybrid aspen, sugar import via the phloem is unlikely due to the cessation of sink activity in para-dormant AXBs, and the accumulation of callose at PD and sieve plates (Supplementary Fig. S4C). Similarly, xylem import of root-produced cytokinin may also not be the initial event given the desiccated state of the AXB and the hard bud scales that prevent evaporation.

Thus, the persistent problem in understanding AXB activation is that the AXM (SAM) of the embryonic shoot must sense a change in the shoot system, as a putative signal must be relayed to it from the stem. We hypothesize that this signal travels via reinvigorated symplasmic connections. This would require first the function of 1,3-β-glucanases that reduce callose at PD and sieve plates (Levy *et al.*, 2007, 2009), opening up symplasmic connections between the stem and AXB. Auxin in the stem could promote removal of dormancy callose in the phloem, as shown for some woody perennials (Aloni *et al.*, 1991; Aloni and Peterson, 1997). In contrast, in the hypocotyl of the herbaceous annual Arabidopsis, auxin can stimulate callose deposition, enabling polar auxin transport during phototropic bending (Han *et al.*, 2014). A role for bud-produced auxin as well as GA-induced 1,3-β-glucanases in AXB activation is supported by the elevated expression of several callose-hydrolyzing α-clade *GH17* members in decapitation-activated AXBs (Fig. 4A), the reduced callose deposits in activated AXBs on internode cuttings (Supplementary Fig. S4D), and the spontaneous branching of transgenic plants overexpressing the α-clade 1,3-β-glucanases (Fig. 5H).

Analysis of *cis*-elements in the promoter region of the decapitation-inducible α-clade *GH17_102* further supports the conjecture that this gene may be under regulation of GA and auxin (Supplementary Table S3). Its promoter has target sequences that are similar to those present in the intron of the *AGAMOUS* gene for *LEAFY* and *WUS* binding (Lohmann *et al.*, 2001). The WUS protein, which moves through PD from the *WUS* domain to the overlying *CLV3* domain (Daum *et al.*, 2014), might facilitate its own movement by recruiting *GH17_102* or another α-clade GH17 enzyme to dilate the PD transport channel. This seems feasible as in Arabidopsis the *WUS* expression domain (Yadav *et al.*, 2009) overlaps with that of a gene homologous to *GH17_102* (Supplementary Fig. S5). Similarly, α-clade genes may play a direct role in AXB activation as the phenotype of *GH17_102* overexpressors display sylleptic-like branches at lower nodes (Fig. 5H). Although the branches in the *GH17_102* lines were short, the phenotype demonstrates that simply increasing PD permeability can overcome an arsenal of inhibitory factors that are present in mature AXBs. The present data suggest that re-functionalization of symplasmic conduits between stem and dwarfed shoot is a determinant of AXB activation and outgrowth (Fig. 7). How this is co-ordinated at the molecular level remains to be established.

### AXB development is accompanied by γ-clade GH17 gene expression

The enhanced expression of γ-clade GH17 genes during AXB development reflects the concurrent accumulation of lipid bodies (Figs 2, 4B). The encoded γ-clade members typically lack known membrane localization signals, and instead they may be stored at lipid bodies, attached to the half-membrane via electrostatic interactions, hydrophobic binding, or accessory molecules (Paul *et al.*, 2014*b*; van der Schoot *et al.*, 2014). They do not play a major role in growth, as decapitation decreases the expression of γ-clade GH17 genes in the proximal AXBs (Fig. 4B). However, as they are accumulating and precociously stored in all AXBs, it seems possible that they function in the initial re-invigoration of symplasmic connections between AXB and stem, prior to outgrowth. The promoter region of the γ-clade member *GH17_44* contains the sugar-responsive *cis*-element SRE that in Arabidopsis is negatively regulated by sugar (Tatematsu *et al.*, 2005). Notably, *GH17_44,* is up-regulated in maturing AXBs that cease sugar import, and down-regulated during AXB activation when sugar import is restarting (Fig. 4B).

The relationship between γ-clade GH17 gene expression and AXB development is not just correlative, as evidenced by overexpression of *GH17_44*, which could induce bud development. Strong overexpressors developed pronounced TBs and AXBs already at the tissue culture stage under LDs (Fig. 5C). The reason is unclear, but possibly overproduction of the enzymes depletes the membrane lipid pool by directing lipid synthesis towards lipid body formation thereby inhibiting elongation and promoting bud formation. Moderate transgenic *GH17_44* lines showed an acrotonic sylleptic branching pattern, with sporadic flushes from the uppermost AXBs (Fig. 6B). These flushes could be due to the tendency to start a TB that temporarily weakens apical dominance, as observed after a brief SD exposure in wild-type plants (Supplementary Fig. S1). The branching of *GH17_44* lines resembles that of tropical trees in which rhythmic meristem activity is accompanied by branching at the end of each flush (Hallé *et al.*, 1978). Our data suggest that transient reduction in symplasmic connectivity and sink activity at the apex of the main stem may contribute to sylleptic branching from young AXBs that are still sinks. What the possible connection is between *GH17_44* and the cytokinin-induced gene *EARLY-BUD BREAK1* (*EBB1*), overexpression of which induces somewhat similar sylleptic branches in *P. deltoides* (Yordanov *et al.*, 2014), remains to be established. It is tempting to speculate that EBB1-like proteins act in the wake of GA-inducible 1,3-β-glucanases that re-functionalize symplasmic input conduits.

### Conclusions

The present findings add to previous work (Rinne *et al.*, 2015) showing that AXB-intrinsic controls are crucial in branching of trees. Novel intrinsic controls include GA pathway genes and GA-regulated GH17-family members that reduce callose depositions at PD and sieve plate pores. Collectively, they enable and facilitate transport and communication between stem and bud.

### Accession numbers

The *P. trichocarpa* gene model identifiers (Tuskan *et al.*, 2006) and/or sequence accessions used for real-time qPCR analysis are listed in Supplementary Table S1.

## Supplementary data

Supplementary data are available at *JXB* online.

Figure S1. Reversal of dormancy development and subsequent branching.

Figure S2. Developmental desiccation of AXBs.

Figure S3. Expression analyses of *GH17_102* and *GH17_79*.

Figure S4. Callose in para-dormant and activated AXBs.

Figure S5. Arabidopsis eFP browser.

Table S1. *Populus trichocarpa* genes, identifiers, and primer pairs.

Table S2. Syllepsis-like branching of *GH17* overexpressors.

Table S3. Analysis of putative promoter regions of *GH17* genes.

Supplementary Data

## Abbreviations:

AXB: axillary bud
AXM: axillary meristem
BMP: bud maturation point
LD: long day
PATS: polar auxin transport stream
PD: plasmodesmata
SAM: shoot apical meristem
SD: short day
TB: terminal bud.
