# Supplementary material for: Axillary buds are dwarfed shoots that tightly regulate GA pathway and GA-inducible 1,3-β-glucanase genes during branching in hybrid aspen
Source: J Exp Bot. 2016 Oct 3;67(21):5975–91. doi: 10.1093/jxb/erw352 (PMC5100014; doi:10.1093/jxb/erw352)
Supplement: Supplementary Data [file supp_67_21_5975__index.html]

Axillary buds are dwarfed shoots that tightly regulate GA pathway and GA-inducible 1,3-β-glucanase genes during branching in hybrid aspen — Axillary buds are dwarfed shoots that tightly regulate GA pathway and GA-inducible 1,3-β-glucanase genes during branching in hybrid aspen — Supplementary Data 

# Axillary buds are dwarfed shoots that tightly regulate GA pathway and GA-inducible 1,3-β-glucanase genes during branching in hybrid aspen

## Supplementary Data

Data files

- supplementary\_figures\_S1\_S5\_tables\_S1\_S3.pdf - Supplementary Data
