# Supplementary material for: Axillary buds are dwarfed shoots that tightly regulate GA pathway and GA-inducible 1,3-β-glucanase genes during branching in hybrid aspen
Source: J Exp Bot. 2016 Oct 3;67(21):5975–91. doi: 10.1093/jxb/erw352 (PMC5100014; doi:10.1093/jxb/erw352)
Supplement: Supplementary Data [file supp_erw352_supplementary_figures_S1_S5_tables_S1_S3.pdf]

Supplementary Figure S1

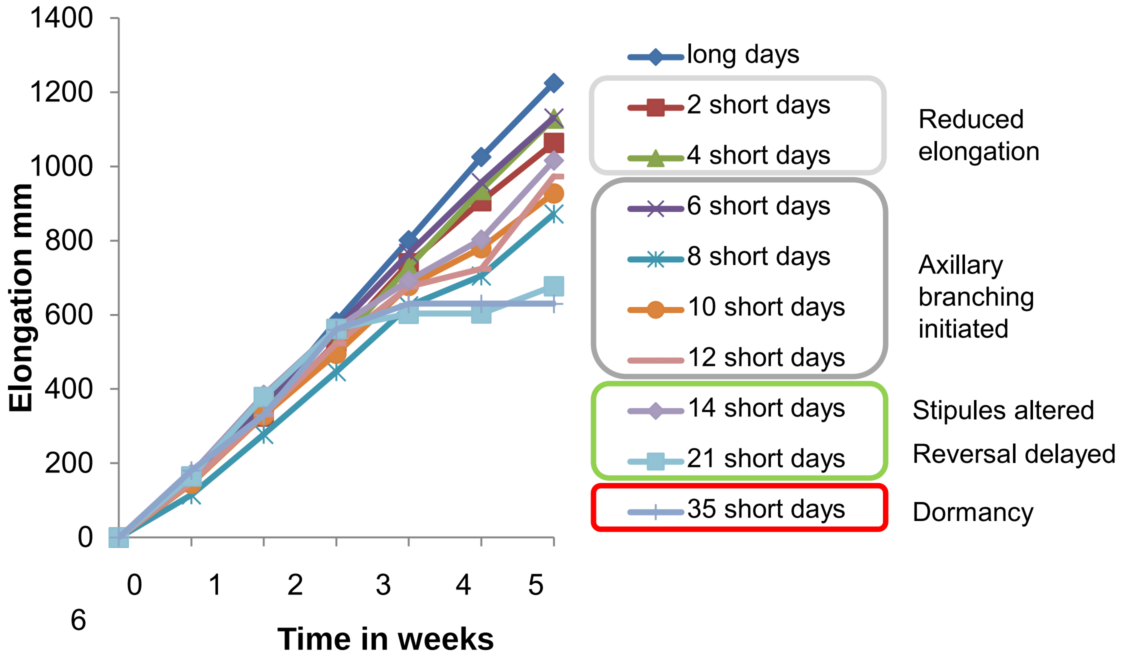

Supplementary Figure S1. Reversal of dormancy development and subsequent branching.

Graph shows growth increments (mm) under a long day photoperiod after pre-exposure to a number of inductive short days. Short day exposure as short as 2-4 cycles was only visible as a slightly reduced elongation (light grey legend box). 6-12 reduced growth gradually more substantially but axillary branching from the top axillary buds was initiated (dark grey legend box). 14-21 days left visible marks in the stipules and reversal to normal growth became increasingly difficult (21 cycles) (green legend box). Reversibility is lost between 21-35 cycles, when terminal buds and AXBs have acquired dormancy (red legend box).

Supplementary Figure S2

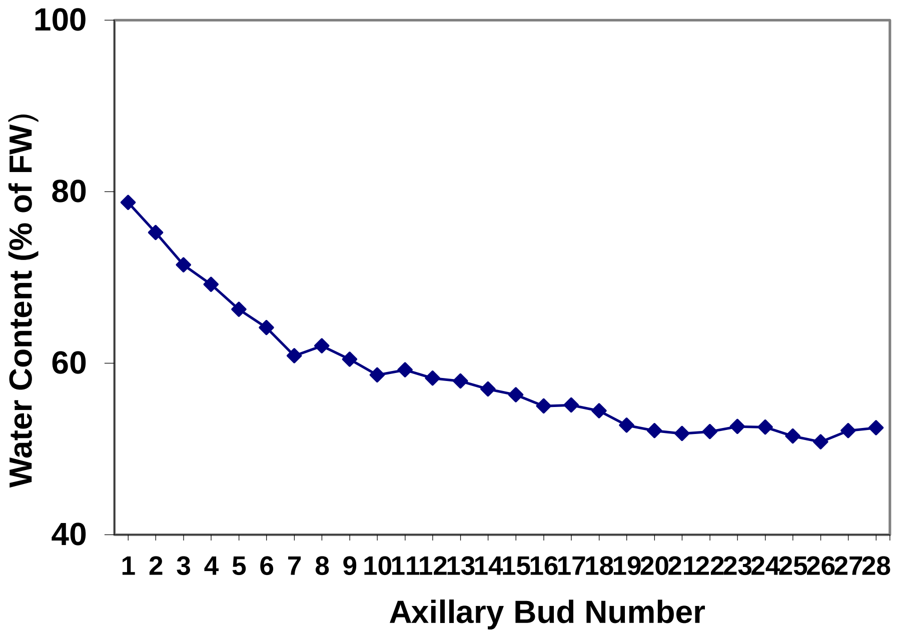

Supplementary Figure S2. Developmental desiccation of AXBs.

Water content (% of fresh weight) of AXBs between positions 1 (top) to 28 (base) in current year shoots grown in long days. Water content in developing AXBs (positions 1-12) decreased by 20%, whereas further maturation (positions 11-20) results in an additional 5% decrease. Values represent pooled samples of three plants.

Supplementary Figure S3

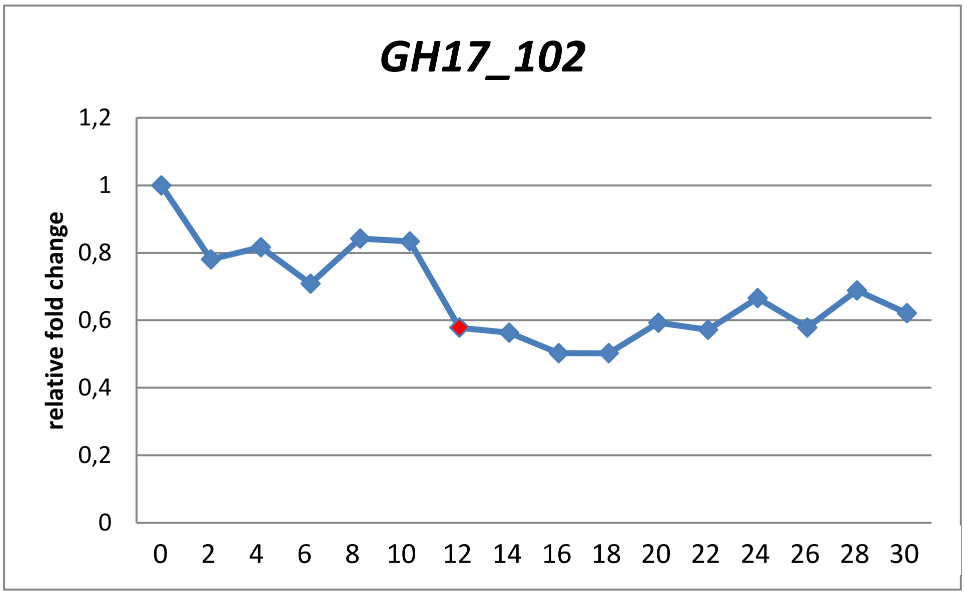

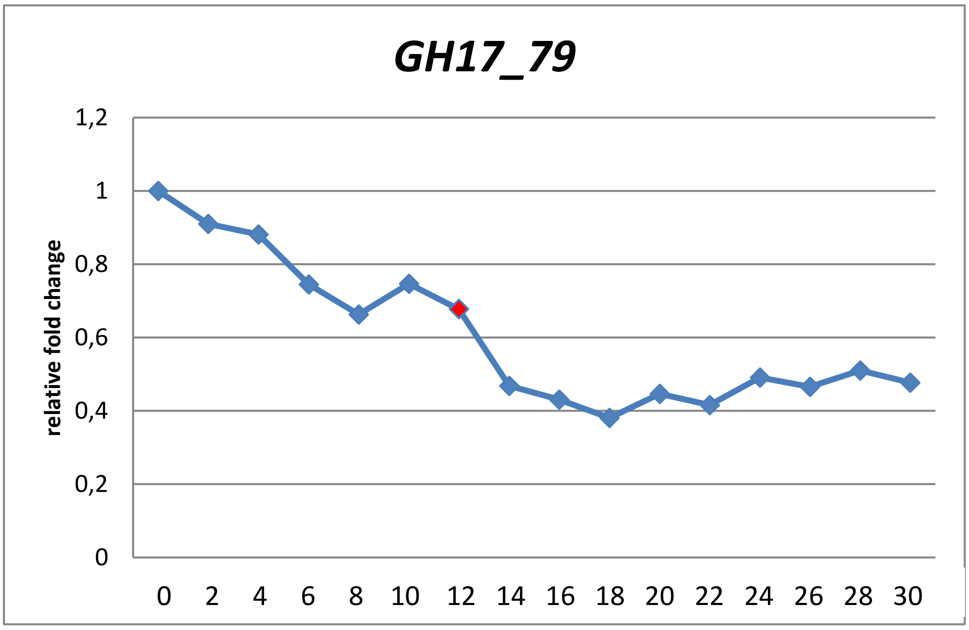

Supplementary Figure S3. Expression analyses of *GH17_102* and *GH17_79*.

Supplementary Figure S4

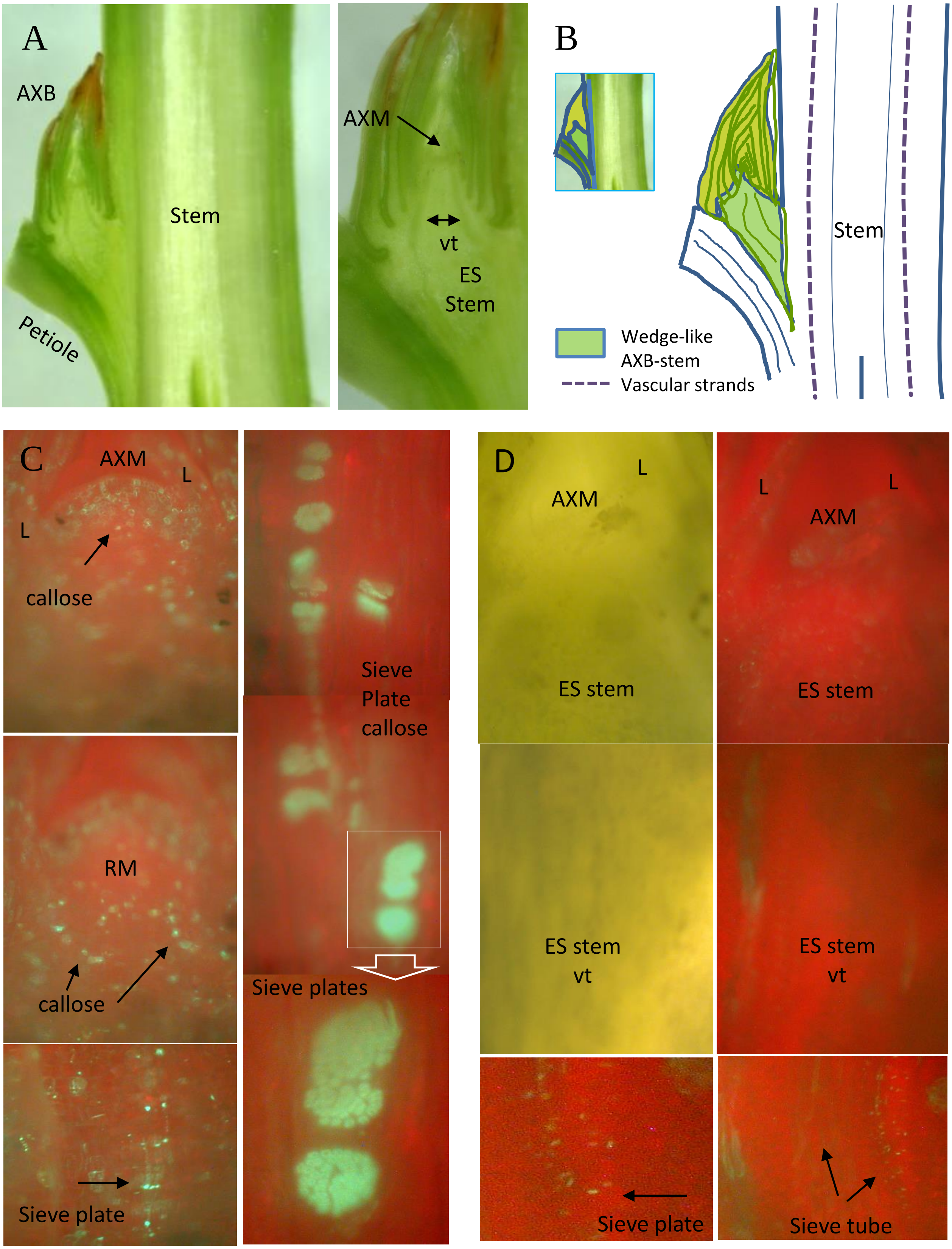

Supplementary Figure S4. Callose in para-dormant AXBs and in activated AXBs.

(A) Longitudinal cross-section through a para-dormant AXB, below the BMP. Detail of AXB to the right. (B) AXB is wedged in between the petiole and the main stem. Vascular strands connect the stem of the embryonic shoot to the main stem. (C) Callose deposition in a para-dormant AXB. Left three panels: Callose at plasmodesmata (PD) in the shoot apical meristem (SAM)(upper panel), callose in pro-vascular traces and at PD in some surrounding cells (middle panel), callose in lower embryonic stem at sieve plates and PD in surrounding cells (lower panel). Right panel: sieve plates with heavy callose depositions. (D) Activated AXB below the BMP, three days post isolation of the AXB-node unit. Strongly diminished callose deposits at both sieve plates and PD in the SAM, embryonic stem, and vascular traces (vt).

Supplementary Figure S5

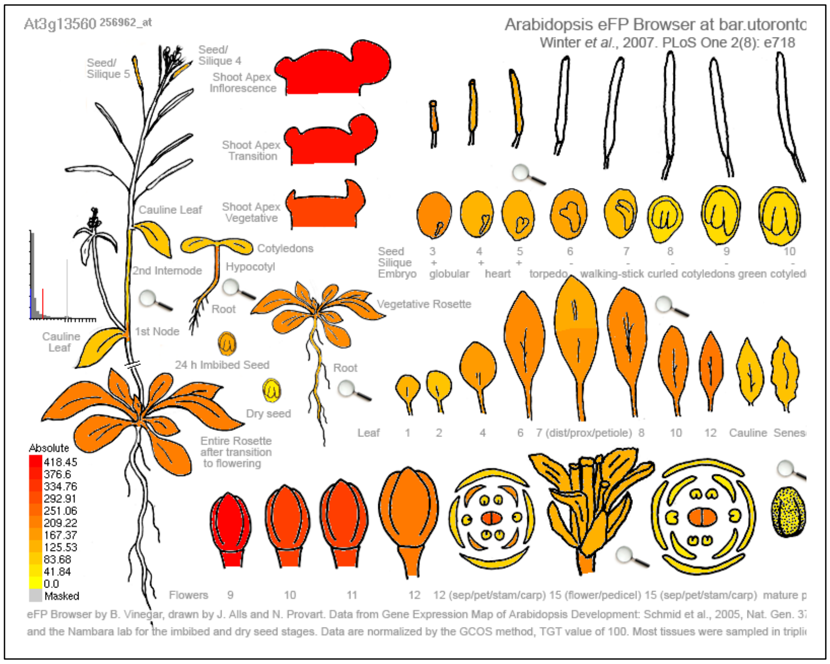

http://bar.utoronto.ca/efp/cgi-bin/efpWeb.cgi?dataSource=Developmental_Map&modelInput=Absolute&primaryGene=At3g13560&secondaryGene=At3g27340&override=&threshold=1583.82&modeMask_low=None&modeMask_stddev=None

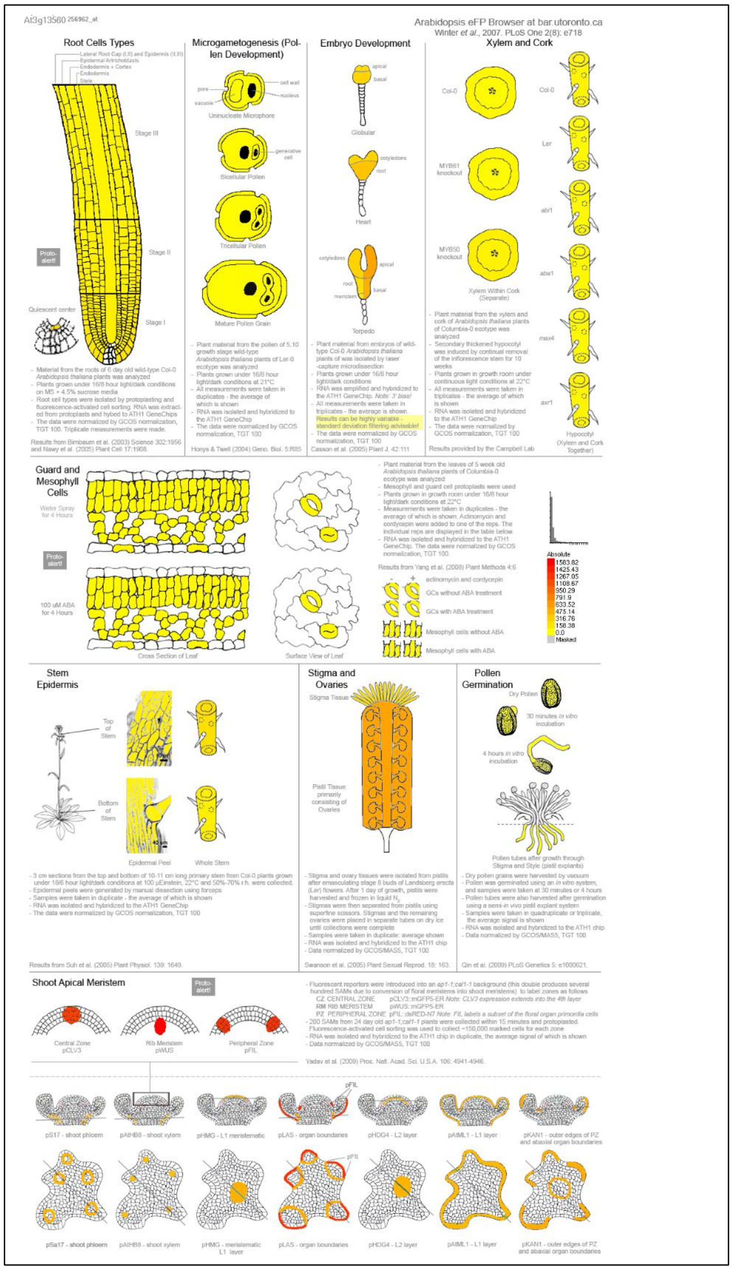

http://bar.utoronto.ca/efp/cgi-bin/efpWeb.cgi?dataSource=Tissue_Specific&modelInput=Absolute&primaryGene=At3g13560&secondaryGene=At3g27340&override=None&threshold=418.45&modeMask_low=None&modeMask_stddev=None

Supplementary Table S1. *P. trichocarpa* genes, identifiers and primer pairs. Gene specific primer sequences for the qPCR analysis were designed using Primer3 (http://frodo.wi.mit.edu/cgi-bin/primer3/primer3_www-cgi).

Protein	Gene abbreviation	Populus trichocarpa v2.0 Locus name	Updated v3.0 Locus name	Primers Forward 5'-3'	Primers Reverse 5'-3'
GH17-genes					
beta-1,3-glucanase	*GH17_33*	POPTR_0001s45320	Potri.001G449100	CAATGCGTATCCCTTTTTCG	GGAAGTTGCTTTTCGGATCA
beta-1,3-glucanase	*GH17_37*	POPTR_0006s04670	Potri.006G048100	AATGCGGCTCTATGATCCAG	CAATCCCTTGAAGGTCAGGA
beta-1,3-glucanase	*GH17_39*	POPTR_0001s26210	Potri.001G255100	AACCTGGGTTCAAGACAACG	CGCATCTCCAGGGTGTACTT
beta-1,3-glucanase	*GH17_44*	POPTR_0010s15270	Potri.010G142800	TGCATTCTCCCCGAATAAAC	GGAAACTGTCGCGTTTTGTT
beta-1,3-glucanase	*GH17_61*	POPTR_0004s21250	Potri.004G202400	GGCTCGGGAATGAGTACCTT	AACACCTCGTTTCCGACAGT
beta-1,3-glucanase	*GH17_65*	POPTR_0011s15560	Potri.011G152400	GCCAAATCAAGGGATTGTTG	TTGCTAATGCAGAATGCACAG
beta-1,3-glucanase	*GH17_79*	POPTR_0002s24930	Potri.002G247900	TCAAAGACACCGACATCCAA	TTAACCCAGTGGTCCGAGAG
PD-callose binbding protein	*GH17_98*	POPTR_0012s06720	Potri.012G065700	ATCAAAATGGCGCTTGTTTC	AAATCACAGGTGCCTTGAGC
beta-1,3-glucanase	*GH17_101*	POPTR_0016s05800	Potri.016G057600	AGAGAGGAACCCCAAAGAAG	AGAAAGATCCCCCAATGTTT
beta-1,3-glucanase	*GH17_102*	POPTR_0001s04360	Potri.001G006500	TGCCATGAACTACCTCCACA	GGAAAGGCCTTGGGATAATG
Gibberellin biosynthesis, catabolism and signaling genes					
GA3 oxidase 1	*GA3ox1*	POPTR_0001s17680	Potri.001G176600	TGGCTCTCCTCTTGAGCATT	AACCATGTCAACCTCCTTGC
GA3 oxidase 2	*GA3ox2*	POPTR_0003s05610	Potri.003G057400	CCCTATCTCGCTCAATCTTTCC	AGTCAAGGTGCTTTTGGTGTAG
GA 20 oxidase 3	*GA20ox3*	POPTR_0005s20660	Potri.005G184400	TCGGATCTCGTTGTGCTAGA	AGTTCCAATATGGCGAAGGA
GA20 oxidase 4	*GA20ox4*	POPTR_0014s06960	Potri.014G073700	TGGCACTCCGTTACTCCTG	AAAGCGAGGGATTTCCTTACT
GA 20 oxidase 6	*GA20ox6*	POPTR_0012s14040	Potri.012G132400	ATTTCGACGCTTTTGTCGTT	GAGATTTTCTTGGCGTTTGG
GA 20 oxidase 7	*GA20ox7*	POPTR_0014s06960	Potri.014G073700	ATGGCACTCCGTTACTCCTG	CCACTGCTCTATGCAAGCAA
GA 20 oxidase 8	*GA20ox8*	POPTR_0015s14030	Potri.015G134600	ATCAAAACCATGCCATCCA	TGGTGTCGAAGAACTTGTGC
GA2 oxidase 1	*GA2ox1*	POPTR_0001s38760	Potri.001G378400	TTCTTCTCATTACCGCTCTCTG	TCTACCCAGCCCACATCAC
GA2 oxidase 2	*GA2ox2*	POPTR_0002s19260	Potri.002G191900	TGCCTTCCAGGTTTTAACGA	GGCAAGACCAGCTGTGGAG
GA2 oxidase 3	*GA2ox3*	POPTR_0004s06380	Potri.004G065000	GGACCTCCTAACCCTTTTGG	TGGGTTTTCCTGAAAAATGG
GA2 oxidase 4	*GA2ox4*	POPTR_0008s10100	Potri.008G101600	AGGTAGGGTTCGGAGAGCAT	GGTAGCGGGATCAGGTGTTA
GA2 oxidase 5	*GA2ox5*	POPTR_0010s15950	Potri.010G149700	AATGGCCTATTTTGCTGCAC	TATCTCCAAGTCGCAGAGCA
GA2 oxidase 6	*GA2ox6*	POPTR_0011s09770	Potri.011G095600	CAAGCCAGCACTTCAACAGT	ATTCCTCACATGCCTTGACC
GIBBERELLIN INSENSITIVE DWARF1	*GID1A*	POPTR_0005s04240	Potri.005G040600	ACCGTGGGACTAGCCTTCTT	ACAACCTCCGAGTTGACAGG
GIBBERELLIN INSENSITIVE DWARF1	*GID1B*	POPTR_0014s13170	Potri.014G135900	GATCATGTTGATCGCACCAC	GTGCTCAAGGGCTTTTCAAG
DELLA	*DE1/RGL1-related*	POPTR_0005s14540	Potri.017G125200	GCAAGTCGAGTCCACGTTATC	AATTCCCGTCAGCCGAAATG
GIBBERELLIN INDUCIBLE PROTEIN 1	*GIP1*	POPTR_0017s11940	Potri.017G083000	TGGACCTGGGAGTCTCAAG	GGAACGCAGAGGCACTTC
Reference gene actin					
ACTIN	*ACT*	POPTR_0001s31700	Potri.001G309500	CGATGCCGAGGATATTCAAC	ACCAGTGTGTCTTGGTCTACCC

Supplementary Table S1.

Supplementary Table S2.

Supplementary Table S2. Syllepsis-like branching of *GH17* overexpressors.

Sylleptic branching of three *GH17_44* and three *GH17_102* overexpression lines. Wild type plants at corresponding age and growth patch as transgenic lines are included for comparison.

Transgenic lines and wild type	[a]Plant height (no of nodes/plant)	Branching event	[a]Nodal positions of branches	Average branch length (cm)
Wild type	226 (70)	none	none	-
GH17_44 OE-9	210 (75)	1st flush<br>2nd flush	37-46<br>8-23	36.3 (±6.4)<br>17.6 (±7.8)
GH17_44 OE-16	198 (64)	1st flush<br>2nd flush	44-47<br>27	32.0 (±17.2)<br>6.0
GH17_44 OE-18	214 (79)	1st flush<br>2nd flush	22-29<br>14-17	13.3 (±6.3)<br>2.4 (±0.2)
Wild type	36 (26)	none	none	-
GH17_102 OE-8	19 (20)	1st flush	11-18	≤2
GH17_102 OE-12	24 (21)	1st flush	9-16	≤2
GH17_102 OE-13	26 (24)	1st flush	10-20	≤2

[a]All height and length values are measured in cm.

[b]Nodal positions are counted from the apex of plant

[c]Values in parenthesis represent minimum to maximum range

Supplementary Table S3.

Supplementary Table S3. Analysis of putative promoter regions of GH17 genes.

Sequences were analyzed using publicly available database of plant cis-acting regulatory DNA elements (http://www.dna.affrc.go.jp/PLACE/) (Higo et al. 1999), and selected DNA elements are presented. Cell wall related GPI-anchored GH17 members are marked with GPI, some possessing an addition callose binding motif (CBM43). The GH17 proteins without signal sequence may associate with lipid bodies (LB).

**Function**	**DNA element**	**Accession no**	**GH17_79 CBM43** No of sites	**GH17_98 CBM43 + GPI** No of sites	**GH17_102 CBM+GPI** No of sites	**GH17_33 GPI** No of sites	**GH17_65 GPI** No of sites	**GH17_37 LB** No of sites	**GH17_39 LB** No of sites	**GH17_44 LB** No of sites	**GH17_101 LB** No of sites	**GH17_61 GPI** No of sites
Auxin response factor	ASF1MOTIFCAMV	S000024	1	--	3	1	--	--	--	--	1	1
	CATATGGMSAUR	S000370	2	6	4	--	--	--	--	--	--	2
	SEBFCONSSTPR10A	S000391	1	1	2	2	1	--	--	--	--	1
Vascular expression	BS1EGCCR	S000352	1	--	1	--	--	--	--	--	--	--
	XYLAT	S000510	--	--	2	--	--	--	1	--	--	--
Gibberellin activity	GARE1OSREP1	S000419	1	1	2	--	--	--	--	--	--	--
	GAREAT	S000439	--	--	1	1	1	2	1	--	--	1
	MYBGAHV	S000181	--	--	1	--	--	2	--	--	--	1
	TATCCACHVAL21	S000416	--	--	1	1	--	--	--	--	--	--
Meristem specific expression	L1BOXATPDF1	S000386	--	1	--	--	--	--	--	2	1	--
	LEAFYATAG	S000432	--	--	1	--	--	--	1	--	--	--
	WUSATAg	S000433	--	--	1	--	--	--	1	--	1	1
Axillary bud outgrowth	SREATMSD	S000470	1	--	--	--	1	2	2	3	1	1
	UP2ATMSD	S000472	--	--	1	--	--	--	--	--	--	--
Phosphate starvation	P1BS	S000459	--	--	4	--	--	--	2	2	--	--
